# Triple negative breast cancer initiating cell subsets differ in functional and molecular characteristics and in γ-secretase inhibitor drug responses

**DOI:** 10.1002/emmm.201302558

**Published:** 2013-08-27

**Authors:** Diana J Azzam, Dekuang Zhao, Jun Sun, Andy J Minn, Prathibha Ranganathan, Katherine Drews-Elger, Xiaoqing Han, Manuel Picon-Ruiz, Candace A Gilbert, Seth A Wander, Anthony J Capobianco, Dorraya El-Ashry, Joyce M Slingerland

**Affiliations:** 1 Braman Family Breast Cancer Institute, Sylvester Comprehensive Cancer Center, University of Miami Miller School of MedicineMiami, FL, USA; 2 Department of Biochemistry and Molecular Biology, University of Miami Miller School of MedicineMiami, FL, USA; 3 Sheila and David Fuente Cancer Biology ProgramMiami, FL, USA; 4 Department of Radiation Oncology, Abramson Family Cancer Research Institute, University of PennsylvaniaPhiladelphia, PA, USA; 5 Molecular Oncology, Department of Surgery, Sylvester Comprehensive Cancer CenterMiami, FL, USA; 6 Department of Medicine, University of Miami Miller School of MedicineMiami, FL, USA

**Keywords:** breast cancer stem cells, GSI, metastasis, Notch1, Sox2

## Abstract

Increasing evidence suggests that stem-like cells mediate cancer therapy resistance and metastasis. Breast tumour-initiating stem cells (T-ISC) are known to be enriched in CD44^+^CD24^neg/low^ cells. Here, we identify two T-ISC subsets within this population in triple negative breast cancer (TNBC) lines and dissociated primary breast cancer cultures: CD44^+^CD24^low+^ subpopulation generates CD44^+^CD24^neg^ progeny with reduced sphere formation and tumourigenicity. CD44^+^CD24^low+^ populations contain subsets of ALDH1^+^ and ESA^+^ cells, yield more frequent spheres and/or T-ISC in limiting dilution assays, preferentially express metastatic gene signatures and show greater motility, invasion and, in the MDA-MB-231 model, metastatic potential. CD44^+^CD24^low+^ but not CD44^+^CD24^neg^ express activated Notch1 intracellular domain (N1-ICD) and Notch target genes. We show N1-ICD transactivates *SOX2* to increase sphere formation, ALDH1+ and CD44^+^CD24^low+^cells. Gamma secretase inhibitors (GSI) reduced sphere formation and xenograft growth from CD44^+^CD24^low+^ cells, but CD44^+^CD24^neg^ were resistant. While GSI hold promise for targeting T-ISC, stem cell heterogeneity as observed herein, could limit GSI efficacy. These data suggest a breast T-ISC hierarchy in which distinct pathways drive developmentally related subpopulations with different anti-cancer drug responsiveness.

## INTRODUCTION

Many cancers appear to be driven by stem-like cells that self-renew, differentiate to yield heterogeneous progeny and survive adverse microenvironments (Dalerba et al, 2007). That these may also initiate metastasis has driven efforts to identify and characterize them. Tumour-initiating stem cell (T-ISC)-enriched populations have been identified in various cancers by discrete surface markers and by their ability to generate tumour spheres and xenograft tumours with high frequency (Frank et al, 2010; Magee et al, 2012; O'Brien et al, 2009). Elegant lineage tracing experiments recently provided compelling evidence for the cancer stem cell model (Chen et al, 2012; Driessens et al, 2012; Schepers et al, 2012). In primary breast cancers, CD44^+^CD24^neg/low^ ESA^+^ cells were enriched for xenograft formation compared to bulk tumour cells (Al Hajj et al, 2003). Aldehyde dehydrogenase 1 (ALDH1) activity also marks breast cancer cells enriched for stem cell properties and those with both ALDH1^+^ and CD44^+^CD24^neg/low^ are most tumourigenic, but infrequent (<1%; Ginestier et al, 2007).

For cancers of breast, pancreas, prostate, head and neck and colon, the T-ISC phenotype consistently includes surface CD44^+^ expression (Al Hajj et al, 2003; Hurt et al, 2008; Li et al, 2007; O'Brien et al, 2007; Prince et al, 2007). CD44 expression has been associated with poor prognosis and metastasis, supporting the idea that stem-like cells generate metastases (Liu et al, 2010; Shipitsin et al, 2007; Yang et al, 2008). In contrast, the relationship between surface CD24 expression and stemness differs between solid tumours. Overexpression of this glycosylated surface protein increases cancer cell proliferation and migration (Aigner et al, 1998). While surface CD24 is observed in subsets of T-ISC from liver (Lee et al, [Bibr b37]), colon (Yeung et al, 2010) and pancreas (Li et al, 2007), the T-ISC phenotype described for primary breast cancers show negative or low level surface CD24 (CD44^+^CD24^neg/low^; Al Hajj et al, 2003). This contrasts with normal mammary progenitors cells, which express CD24 (Pece et al, 2010; Spike et al, 2012). It is noteworthy that CD24^+^ cells are increased in metastatic compared to primary breast cancers (Shipitsin et al, 2007).

Populations surviving chemotherapy and radiation appear to be enriched for T-ISC (Calcagno et al, 2010; Li et al, 2008; Tanei et al, 2009), possibly due to membrane transporters, greater quiescence and enhanced DNA repair, permitting T-ISC regeneration (Frank et al, 2010; O'Brien et al, 2009). Thus, identification and interdiction of T-ISC specific pathways may permit greater anti-cancer treatment efficacy. To date, the difficulty of isolating viable T-ISC from solid tumours in sufficient quantity to permit their molecular characterization has limited development of T-ISC-directed therapies that circumvent drug resistance or induce differentiation. Breast cancer cell lines have been shown to contain T-ISC analogous to those in primary breast cancers, permitting isolation of the large numbers of T-ISC required for functional characterization (Charafe-Jauffret et al, 2009; Fillmore & Kuperwasser, 2008).

Stem-like cell subsets within a cancer may vary not only in their self-renewal potential, but also in their ability to successfully engage different metastatic niches. While T-ISC or a sub-population thereof have been broadly posited as giving rise to metastasis, relatively few experimental models have addressed this directly. ALDH1^+^CD44^+^CD24^neg^ subpopulations in breast cancer lines yielded more xenograft metastasis than ALDH1^-^CD44^low/−^CD24^+^ (Croker et al, 2008), but metastatic potential was not limited to the very low minority ALDH1^+^ population. While increasing data indicate the presence of functional heterogeneity within T-ISC enriched populations in other tumours (Hermann et al, 2007; Lee et al, [Bibr b37]; Pang et al, 2010; Yang et al, 2008), identification and characterization of mammary T-ISC subsets that consistently metastasize or that mediate therapy resistance presents a challenge.

The present study was undertaken to identify discrete subsets among T-ISC of the most deadly form of breast cancer: that lacking estrogen and progesterone receptors and HER2 amplification (so called triple negative—hereafter TNBC). We postulated that, as for normal stem cells, primary TNBC-derived cultures and immortal lines might exhibit an aberrant T-ISC hierarchy with precursor/progeny populations that differ in molecular pathways conferring self-renewal, tumourigenicity and metastatic potential.

Here we demonstrate a hierarchical relationship between distinct subsets within CD44^+^CD24^neg/low^ subpopulations from a TNBC line and from two TNBC patient-derived dissociated tumours (DTs). The minority CD44^+^CD24^low+^ population shows greater sphere formation and gives rise to CD44^+^CD24^neg^ progeny. In contrast, cells arising from CD44^+^CD24^neg^ are exclusively CD44^+^CD24^neg^ both in 2D culture and in spheres. CD44^+^CD24^low+^ are enriched for embryonic stem cell (ES) and metastatic gene expression signatures, tumour sphere, soft agar colony forming and tumour forming cells compared to CD44^+^CD24^neg^, and in the MDA-MB-231 model show greater metastatic potential. In CD44^+^CD24^low+^ cells, Notch1 was shown to directly transactivate *SOX2* to drive self-renewal. Although Notch has been previously implicated in breast cancer stem cell self-renewal (Harrison et al, 2010; McGowan et al, 2011; Sansone et al, 2007) the CD44^+^CD24^neg^ T-ISC sub-population was unaffected by Notch inhibition in 2D culture, sphere and xenograft assays, revealing a heretofore unappreciated heterogeneity in GSI responsiveness in T-ISC.

## RESULTS

### A subset of TNBC lines and patient-derived dissociated tumours contain two distinct stem cell populations

The CD44^+^CD24^neg/low^ breast cancer population was shown to be enriched for cancer initiating stem cells (Al Hajj et al, 2003). Here we investigated the potential existence within this phenotype of subsets with differing self-renewal and tumour initiating abilities. Surface CD44 and CD24 expression were assayed in established breast cancer lines and in seven patient-derived TNBC dissociated tumour cultures (DTs). DTs were used at early passage and their morphologic and molecular characteristics, including gene expression profiling, resemble the original patient tumours from which they were derived (Bayliss et al, 2007). Although all DTs were derived from primary TNBC, their gene expression profiles vary: DT-28 has a basal/epithelial phenotype by PAM-50; DT-22 and DT-25 (as for MDA-MB-231) are basal; DT16 is luminal B and DT-13 localizes to the HER2^+^ expression profile.

**Figure 1:**
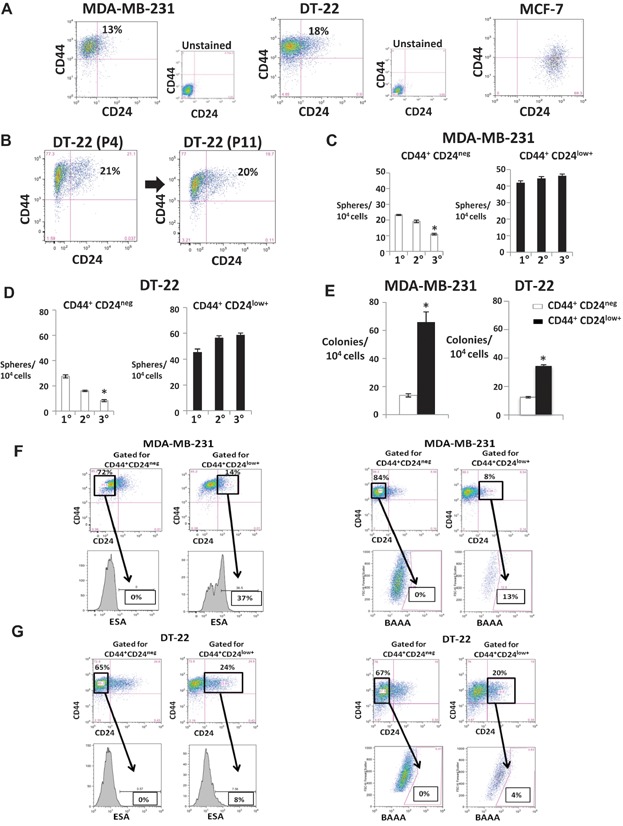
CD44^+^CD24^low+^ and CD44^+^CD24^neg^ population characteristics **A.** CD44 and CD24 in MDA-MB-231, DT-22 and MCF7. Unstained controls are shown.**B.** Surface expression of CD44 and CD24 in DT-22 at passage four (P4) was similar to that at passage 11 (P11).**C,D.** Mean ± SEM serial mammospheres formed/10^4^ cells seeded from sorted CD44^+^CD24^low+^ and CD44^+^CD24^neg^ from MDA-MB-231 (*p = 0.0003) (C) and DT-22 (*p = 0.0001 Student's *t*-test) (D).**E.** Mean ± SEM soft agar colonies from MDA-MB-231 (*p = 0.00024) and DT-22 (*p = 0.0016).**F,G.** ESA^+^ and ALDH1^+^ are detected in a minority of CD44^+^CD24^low+^ but not in CD44^+^CD24^neg^ populations. CD24 and CD44 were assayed together with either ESA or Aldefluor assays as described. Cells gated CD44^+^CD24^neg^ and CD44^+^CD24^low+^ from MDA-MB-231 (F) and DT-22 (G) were assayed for percentage of surface ESA (left) and percentage of ALDH1^+^ cells (right).

Notably, most of the 14 estrogen receptor (ER) negative lines and DTs assayed show a high percent of CD44^+^CD24^neg/low^ cells, while ER positive lines (as described (Charafe-Jauffret et al, 2009; Fillmore & Kuperwasser, 2008)), vary in CD44 staining and have higher CD24 than most ER negative cultures (Fig 1A (right) and Supporting Information Fig S1). Interestingly, a minority of TNBC lines and DTs tested (BT-20, BT-549 and DT-28), showed higher CD24 expression and few if any CD24 negative cells (Supporting Information Fig S1). Thus, the most common CD44^+^CD24^neg/low^ phenotype of TNBC investigated herein is not the only pattern observed within TNBC.

MDA-MB-231, DT-22 and DT-25 (Fig. 1 and Supporting Information Fig S1) were representative of the majority of TNBC cultures assayed with over 90% CD44^+^ cells, comprising a major population of CD44^+^CD24^neg^ cells (>80%) and a minor CD44^+^ population with low level surface CD24 positivity or CD44^+^CD24^low+^ (<20%) cells (see Fig 1A). Failure to stain surface CD24, or CD24-negativity (CD24^neg^), was defined by the gate set from unstained controls. While most TNBC showed a subset of cells with low level surface CD24 positivity (CD24^low+^) the extent of CD24 staining was considerably less than that in ER positive lines (Fig 1A, right). Admixture of MCF-7 and MDA-MB-231 shows how these differ in CD24 staining and identifies the subset defined as CD24^low+^ in TNBC lines (see Supporting Information Fig S1D).

The expression of CD44 and CD24 markers in the DT cultures was highly stable over multiple passages, as was their growth rate. Notably the proportion of CD44^+^CD24^low+^ cells in passage four DT-22 was similar to passage 11 (representative data, Fig 1B). Likewise, CD44 and CD24 expression was similar in DT-25 at passages three and nine (Supporting Information Fig S2A). Potential differences in stem cell characteristics of CD44^+^CD24^neg^ and CD44^+^CD24^low+^ TNBC subpopulations were further investigated.

### CD44^+^CD24^low+^ cells are more spherogenic and contain ESA^+^ and ALDH1^+^ subpopulations

A property of stem cells is the ability to generate spheres. CD44^+^CD24^neg^ and CD44^+^CD24^low+^ cells were isolated by flow sorting and plated at single cell density for sphere formation. While both formed mammospheres, the proportion of sphere forming cells was greater in CD44^+^CD24^low+^ than CD44^+^CD24^neg^ cells in MDA-MB-231, DT-22 and DT-25. Upon serial passage, the proportion of sphere forming cells in CD44^+^CD24^low+^ remained higher than CD44^+^CD24^neg^, with no attenuation of secondary and tertiary mammosphere formation. In contrast, sphere formation by CD44^+^CD24^neg^ cells decreased progressively on serial mammosphere plating in both DT cultures and in MDA-MB-231 (Fig 1C and D, Supporting Information Fig S2B). Notably, clonogenicity in soft agar, a hallmark of cancer forming cells was also enhanced in CD44^+^CD24^low+^ cells compared to CD44^+^CD24^neg^ in MDA-MB-231 and DT22 (Fig. 1E).

Surface epithelial specific antigen + (ESA, also known as EpCAM; Al Hajj et al, 2003) and ALDH1^+^ populations (Ginestier et al, 2007) have also been shown to define minority populations enriched for T-ISC. In DT-22 and MDA-MB-231, the proportions of ALDH1^+^ and of ESA^+^ cells were low (1–2% and 2–5%, respectively) (Supporting Information Fig S2C). To test how ESA^+^ and ALDH1^+^ may relate to CD44 and CD24, cells were gated for ESA or ALDH1 positivity and then analysed for CD44/CD24. In both cell types, the majority of cells gated as positive for ESA (82–92%) were CD44^+^CD24^low+^ (Supporting Information Fig S2D). When gated by CD44/CD24 status, CD44^+^CD24^neg^ cells were ESA negative in both DT-22 and MDA-MB-231. In contrast, one-third of CD44^+^CD24^low+^ MDA-MB-231 cells were ESA^+^, with the percent a little lower in DT-22 (representative data Fig 1F and G left). Similarly, gating on ALDH1^+^ cells showed >95% were CD44^+^CD24^low+^ (Supporting Information Fig 2D). When gated by CD44/CD24 subsets, ALDH1^+^ cells were not detected in CD44^+^CD24^neg^ cells, while a minority of CD44^+^CD24^low^ cells (4–13%) consistently showed ALDH1 positivity in both lines (Fig 1F and G right). Thus, even within the CD44^+^CD24^low+^ there is discernible phenotypic heterogeneity with respect to ESA and ALDH1 positivity.

### CD44^+^CD24^low+^ cells self-renew and give rise to CD44^+^CD24^neg^ progeny

**Figure 2:**
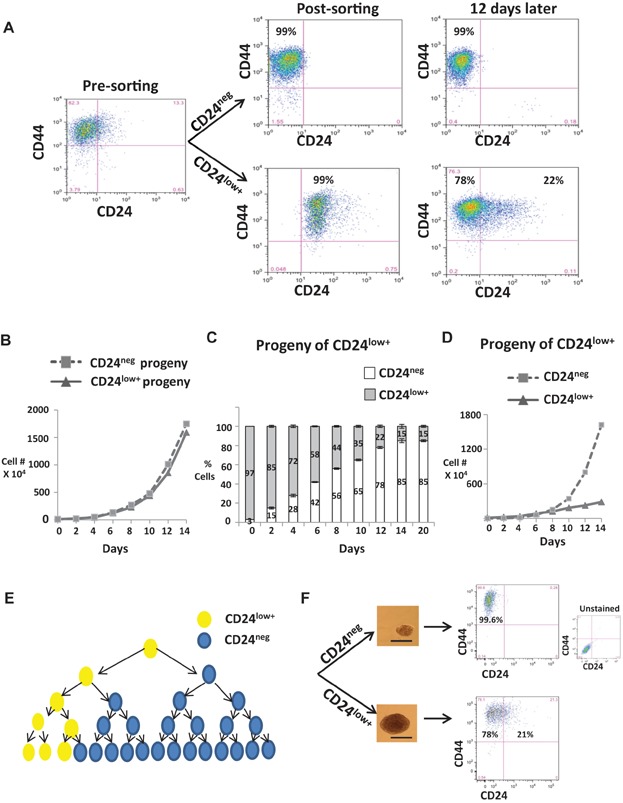
CD44^+^CD24^low+^ give rise to both CD44^+^CD24^low+^ and CD44^+^CD24^neg^ progeny while CD44^+^CD24^neg^ yield only CD44^+^CD24^neg^ CD44^+^CD24^low+^ or CD44^+^CD24^neg^ MDA-MB-231 were sorted and 100,000 cells cultured over 21 days. CD44 and CD24 analysis before and 12 days after sorting.Population growth from sorted CD44^+^CD24^low+^ and CD44^+^CD24^neg^.Proportions of CD44^+^CD24^low+^ or CD44^+^CD24^neg^ cells arising from CD44^+^CD24^low+^ cells.Growth curves of progeny arising from CD44^+^CD24^low+^ over time. Findings validated in DT-22 and DT-25, see in Supporting Information Fig S3.Model depicting largely asymmetric division of CD44^+^CD24^low+^ cells generating mostly CD44^+^CD24^neg^ progeny with a modest increase in CD44^+^CD24^low+^ over time.CD44 and CD24 analysis of dissociated spheres initiated by single isolated CD44^+^CD24^low+^ or CD44^+^CD24^neg^ cells. Scale bar = 75 µm.

To investigate a potential lineage relationship between CD44^+^CD24^low+^ and CD44^+^CD24^neg^ populations, these were sorted from MDA-MB-231, DT-22 and DT-25, re-plated into culture and population growth and surface markers monitored over 3 weeks. Growth curves showed purified CD44^+^CD24^neg^ cells proliferate exponentially but generated only CD44^+^CD24^neg^ cells (MDA-MB-231 data in Fig 2; for DT-22 and DT-25 see Supporting Information Fig S3). In contrast, an over 98% pure CD44^+^CD24^low+^ population gave rise progressively to CD44^+^CD24^neg^ cells, yielding a steady state population at 2–3 weeks of largely CD44^+^CD24^neg^ (85%) with a minority of CD44^+^CD24^low+^ (15%) similar to unsorted MDA-MB-231 cells (Fig 2A, C and D). The cumulative population growth from 100,000 sorted cells of each subpopulation was identical (Fig 2B). However, while CD44^+^CD24^neg^ grew exponentially over at least seven population doublings, CD44^+^CD24^low+^ progeny showed two patterns: CD44^+^CD24^low+^ cell numbers increased arithmetically but generated exponentially growing CD44^+^CD24^neg^ progeny (Fig 2D). These data are compatible with a model in which CD44^+^CD24^low+^ undergo largely asymmetric division, with a modest CD44^+^CD24^low+^ population increase due to less frequent symmetric divisions (Fig 2D and E). The ability of CD44^+^CD24^low+^ to generate both cell types, while CD44^+^CD24^neg^ cells generated only like progeny was also observed in two independent primary dissociated tumour cultures, DT-22 and DT-25 (Supporting Information Fig S3). That CD44^+^CD24^low+^ breast cancer cells gave rise to CD44^+^CD24^neg^ is consistent with Meyers et al (2009). In contrast, our CD44^+^CD24^neg^ cells generated only CD44^+^CD24^neg^ progeny in 2D culture and in spheres (below).

That CD44^+^CD24^low+^ cells give rise to both cell types, while CD44^+^CD24^neg^ progeny are uniquely CD44^+^CD24^neg^ is further supported by sphere assays. Spheres generated from single cells seeded from either CD44^+^CD24^low+^ or CD44^+^CD24^neg^ cells were dissociated and analysed. Strikingly, CD44^+^CD24^neg^-initiated mammospheres contained only CD44^+^CD24^neg^ cells even over three serial sphere assays, while CD44^+^CD24^low+^-initiated mammospheres contained both CD44^+^CD24^low+^ and CD44^+^CD24^neg^ cells (Fig 2F). Thus, both in 3D and 2D culture, CD44^+^CD24^low+^ cells can self-renew and produce CD44^+^CD24^neg^ progeny, while CD44^+^CD24^neg^ have a restricted phenotype.

### CD44^+^CD24^low+^ have a higher proportion of tumour initiating cells than CD44^+^CD24^neg^

**Figure 3:**
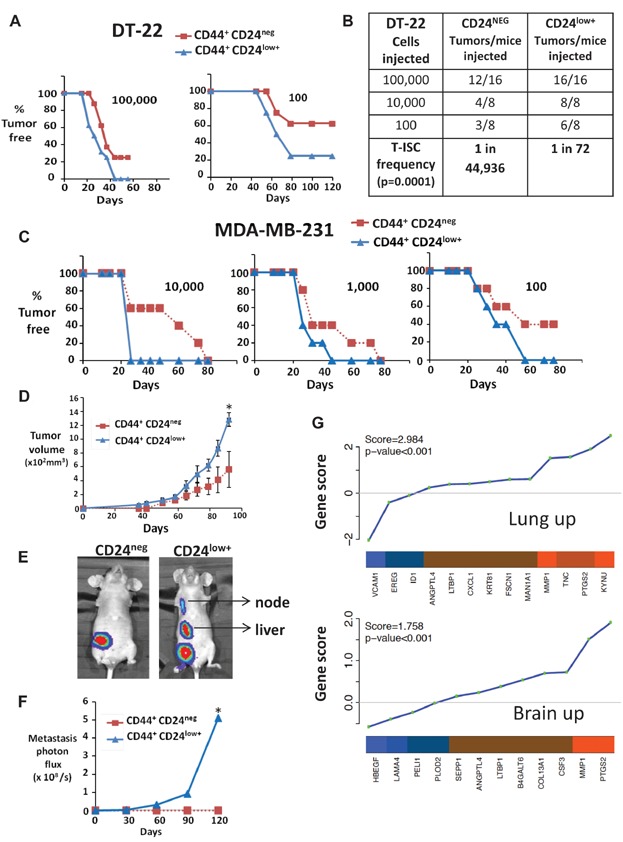
CD44^+^CD24^low+^ generate orthotopic tumours with higher frequency and shorter latency Sorted CD44^+^CD24^neg^ or CD44^+^CD24^low+^ from MDA-MB-231 and DT-22 cells were injected orthotopically as described. Tumour formation from indicated numbers of sorted CD44^+^CD24^neg^ (red) or CD44^+^CD24^low+^ (blue) DT22 cells is graphed over time.Calculated T-ISC frequency from limiting dilution assays with DT22 CD44^+^CD24^neg^ and CD44^+^CD24^low+^.Tumours from 10,000, 1000 and 100 sorted CD44^+^CD24^neg^ (red) or CD44^+^CD24^low+^ (blue) MDA-MB-231 cells.Mean MDA-MB-231 xenograft volume from 10,000 injected CD44^+^CD24^neg^ (red) or CD44^+^CD24 (blue) cells (means graphed ± SEM, *p = 0.019, comparative analysis of growth curves).Primary tumours (1 cm diameter) were excised and metastasis monitored by IVIS. Representative IVIS images are shown.Bioluminescence (photons × 10^8^/s) of metastatic tumour burden from CD44^+^CD24^low+^ tumours and CD44^+^CD24^neg^ is graphed (mean graphed ± SEM, *p = 0.001, comparative analysis of growth curves).Gene set analysis (GSA) shows preferential expression of lung and brain metastasis signatures in CD44^+^CD24^low+^ versus CD44^+^CD24^neg^. Shown are ordered gene scores for each gene in the line plot and the average fold change in the heatmap (orange = high expression in CD44^+^CD24^low+^ and blue = low); enrichment score and *p*-value shown in upper left (see also bone mets signature and similar GSA for sorted DT-22 in Supporting Information Fig S4).

While the ability to form xenograft tumours in immunocompromised mice may underestimate human T-ISC frequency, it is a key functional assay. The tumourigenic potential of CD44^+^CD24^low+^ and CD44^+^CD24^neg^ subpopulations from luciferase tagged MDA-MB-231 and DT-22 was titrated by limiting dilution orthotopic xenograft assays. Different numbers of CD44^+^CD24^low+^ or CD44^+^CD24^neg^ cells (100,000, 10,000 or 100 cells for DT-22 and 500,000, 100,000, 10,000, 1000 or 100 cells for MDA-MB-231) were injected into BalbC nude mice. For each number injected, CD44^+^CD24^low+^ cells generated tumours with shorter latency (Fig 3A and C). The reduced tumour initiating ability of CD44^+^CD24^neg^ DT-22 was reproducibly evident at several different cell numbers injected (Fig 3A and B). One hundred CD44^+^CD24^low+^ DT-22 cells formed tumours in only 3/8 mice (37.5%) with no further tumours emerging over the next ten months of observation (Fig 3A). The T-ISC frequency calculated using the L-Calc software for limiting dilution analysis as in (Korkaya et al, 2012) was significantly higher in the CD44^+^CD24^low+^ population (1 in 72) as compared to CD44^+^CD24^neg^ population (1 in 44,936), two tailed *p* = 0.0001 (Fig 3B). Because all of the 100 CD44^+^CD24^low+^ MDA-MB-231 cell injections yielded tumours, the L-Calc software could not be used to calculate T-ISC frequency. However it is noteworthy that only 60% of 100 cell CD44^+^CD24^neg^ -injected animals formed tumours, suggesting that T-ISC frequency may also be decreased in this subset in MDA-MB-231 as well (Fig 3C, right). CD44^+^CD24^low+^-initiated tumours grew more rapidly than those from CD44^+^CD24^neg^ cells (data for MDA-MB-231 10,000 cell injections, Fig 3D; data for DT-22 100,000 cell injections, Fig 6G). Thus, the T-ISC frequency is higher in the CD44^+^CD24^low+^ population than in CD44^+^CD24^neg^, consistent with their greater colony and sphere forming abilities.

### Metastasis observed only from CD44^+^CD24^low+^-generated MDA-MB-231 tumours

Having shown a greater frequency of sphere and tumour forming cells in CD44^+^CD24^low+^ than CD44^+^CD24^neg^ populations, and a potential precursor–progeny relationship between the two, we next assayed metastatic potential. Animals were monitored by *in vivo* imaging system (IVIS) after excision of orthotopic MDA-MB-231 tumours for subsequent metastasis. While all primary tumours were excised at 1 cm, only CD44^+^CD24^low+^ derived tumours showed metastatic potential. CD44^+^CD24^low+^ tumours metastasized frequently to lymph nodes, liver and/or spleen (10/19 animals). In contrast, none of the CD44^+^CD24^neg^ tumours formed metastasis (0/19 animals), (representative images and graphed bioluminescence from metastasis, Fig 3E and F). CD44^+^CD24^low+^ orthotopic tumours also yielded extensive pulmonary micrometastasis, not detected by IVIS, that were not observed in animals bearing CD44^+^CD24^neg^-generated tumours. Thus, CD44^+^CD24^low+^ cells not only exhibit greater features of stem cells *in vitro*, these also exhibit greater metastatic frequency *in vivo*.

In the DT-22 model, injection of 100,000 cells of each cell type failed to yield metastasis when tumours were excised at 1 cm and animals followed for a further 10 months. This model proved not to generate metastasis in either nude mice or in NOD-SCID mice, a property not known at the outset of the year-long xenograft experiments executed herein. Thus differences in the metastatic ability of the two cell types assayed could not be evaluated in this model.

### CD44^+^ CD24^low+^ cells preferentially express metastasis signatures

MDA-MB-231 variant lines with discrete metastatic tissue tropisms have been used to define gene expression signatures for lung, bone or brain metastasis (Bos et al, 2009; Kang et al, 2003; Minn et al, 2005). In particular, the 18-gene lung and 17-gene brain metastasis signatures are clinically relevant because they can be observed in primary breast cancers and predict metastatic outcome (Bos et al, 2009; Minn et al, 2005). Gene expression profiles of CD44^+^CD24^low+^ and CD44^+^CD24^neg^ populations were compared after cell sorting. Expression of genes from all three metastastic gene signatures is enriched in CD44^+^CD24^low+^ cells from MDA-MB-231 compared to CD44^+^CD24^neg^ (see Fig 3G and Supporting Information Fig S4A–C). Similarly, CD44^+^CD24^low+^ cells from DT-22 showed enrichment of lung and bone metastasis signature genes (Supporting Information Fig S4D and E).

### CD44^+^ CD24^low+^ show activated Notch pathways and higher embryonic stem cell gene expression

**Figure 4:**
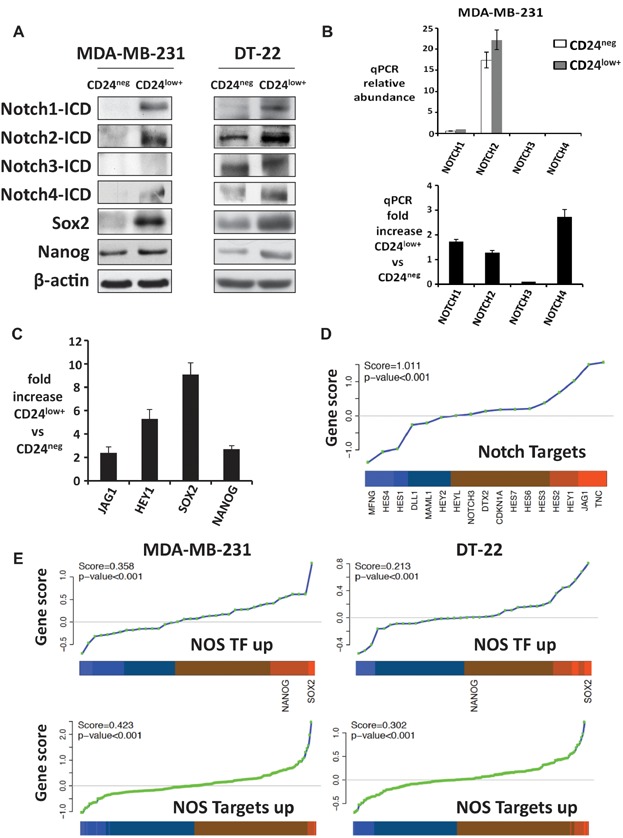
Notch1-ICD, Notch2-ICD, Notch4-ICD and embryonic transcription factors are preferentially expressed in CD44^+^CD24^low+^ CD44^+^CD24^neg^ (CD24^neg^) and CD44^+^CD24^low+^ (CD24^low+^) subpopulations of MDA-MB-231 and DT-22 cells were flow sorted. Western blots.Relative abundance of Notch 1, 2, 3 and 4 (top) expression by qPCR and mean fold increase in CD24^low+^ versus CD24^neg^ ± SEM (bottom) are shown for MDA-MB-231.Notch target gene expression is enriched in MDA-MB-231 CD44^+^CD24^low+^ cells.GSA shows CD44^+^CD24^low+^ preferentially express Notch target genes defined by GSI washout.GSA shows CD44^+^CD24^low+^ preferentially express NOS-targets and NOS-TFs. The ordered scores for each gene in the line plot and the average fold change in the heatmap are shown (orange indicates high in CD44^+^CD24^low+^ and blue, low) with enrichment scores and *p* values (see similar GSA for ES-TFs for both lines and NOS-targets, and NOS-TFs in sorted DT-22 in Supporting Information Fig S5).

Pathways driving embryonic stem cell self-renewal have been implicated in mammary cancer stem cell maintenance (Liu et al, 2006). In both MDA-MB-231 and DT-22, greater Notch pathway activation was observed in CD44^+^CD24^low+^ cells, with increased protein levels of the intracellular domains N1-ICD, N2-ICD and N4-ICD (Fig 4A). CD44^+^CD24^low+^ cells also showed higher *NOTCH1*, *NOTCH2* and *NOTCH4* expression levels than in CD44^+^CD24^neg^ (Fig 4B) and increased *JAG1* and *HEY1* expression (MDA-MB-231, Fig 4C). Interestingly, in sorted MDA-MB-231 the relative expression levels of *NOTCH1* and *NOTCH2* genes were higher than those of *NOTCH3* and *NOTCH4* as assayed by q-PCR (MDA-MB-231, Fig 4B, top). To confirm Notch pathway activation, a profile of genes upregulated following release from gamma-secretase inhibition was characterized by gene expression arrays. CD44^+^CD24^low+^ cells from MDA-MB-231 showed significant overexpression of this Notch activation signature compared to CD44^+^CD24^neg^ (Fig 4D).

Embryonic transcription factors Sox2 and Nanog are required for embryonic stem cell (ES) self-renewal (Li, 2010). Both were increased in CD44^+^CD24^low+^ versus CD44^+^CD24^neg^ cells (Fig 4A). It is postulated that ES transcription factors (ES-TFs) contribute to T-ISC self-renewal, but few studies have demonstrated ES transcriptional program changes in tumour initiating cells. ES-TF upregulation in CD44^+^CD24^low+^ cells was validated by expression profiling in both MDA-MB-231 and DT22. Expression profiles characteristic of human ES (Assou et al, 2007), and genes whose promoters are bound and activated by Nanog, Oct4 and Sox2 (NOS targets) in ES, and the subset of NOS targets encoding transcriptional factors (NOS TFs; Ben-Porath et al, 2008; Boyer et al, 2005) were all significantly enriched in CD44^+^CD24^low+^ compared to CD44^+^CD24^neg^ cells from both MDA-MB-231 and the DT-22 culture (Fig 4E, Supporting Information Fig S5A–F and Table S1). Thus, ES transcription programs are preferentially upregulated in the CD44^+^CD24^low+^ subpopulation over CD44^+^CD24^neg^.

### Self-renewal in CD44^+^CD24^low+^ involves Notch1 mediated Sox2 activation

**Figure 5:**
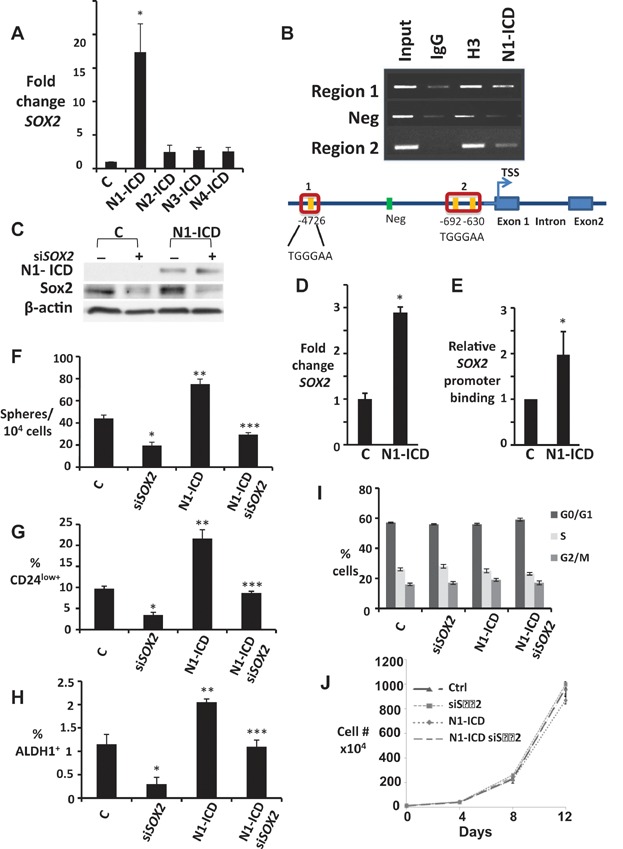
Notch1-mediated Sox2 activation governs stem cell-like phenotype of CD44^+^CD24^low+^ cells **A.** *SOX2* qPCR in HC-11 cells ± N1-ICD, N2-ICD, N3-ICD or N4-ICD overexpression, graphed as fold change versus parental controls, normalized to HPRT. *p = 0.018.**B.** ChIP shows N1-ICD and control histone H3 binding to indicated CSL binding sites in murine *SOX2* promoter in HC-11 (top). Schematic of murine *SOX2* promoter (bottom).**C–E.** Controls or N1-ICD overexpressing MDA-MB-231 cells were assayed for N1-ICD and Sox2 by Western (C) *SOX2* expression by qPCR (*p = 0.006) (D) and relative binding of N1-ICD to *SOX2* promoter (E) and means graphed ± SEM.**F–H.** Effects of si*SOX2* were assayed in MDA-MB-231 ± N1-ICD overexpression on spheres formed/10,000 plated cells (F), % of CD44^+^CD24^low+^ (G) and % ALDH1^+^ cells (H). Graphs show mean ± SEM; For (F), ***p = 0.019, **p = 0.032 and *p = 0.026; for (G), ***p = 0.0049, **p = 0.0048 and *p = 0.0007 and for (H), ***p = 0.04, **p = 0.03 and *p = 0.01 (Student's *t*-test compared to control, C).**I.** Cell cycle profiles of si*SOX2* in MDA-MB-231 ± N1-ICD overexpression.**J.** Proliferation curves are unchanged by siSOX2 in MDA-MB-231 cells ± N1-ICD overexpression.

Since both NOTCH pathway activation and Sox2 levels were upregulated in CD44^+^CD24^low+^ compared to CD44^+^CD24^neg^, the relationship between them and their importance to sphere-forming ability, a proxy assay of self-renewal was further investigated. Notably, N1-ICD transduction into immortalized HC-11 mammary epithelial cells caused >20-fold increase in *SOX2* expression while other Notch isoforms did not (Fig 5A). The *SOX2* promoter in human and murine cells contains multiple Notch consensus motifs. ChIP analysis demonstrated N1-ICD binding to two different *SOX2* promoter sites in HC-11 (Fig 5B).

**Figure 6:**
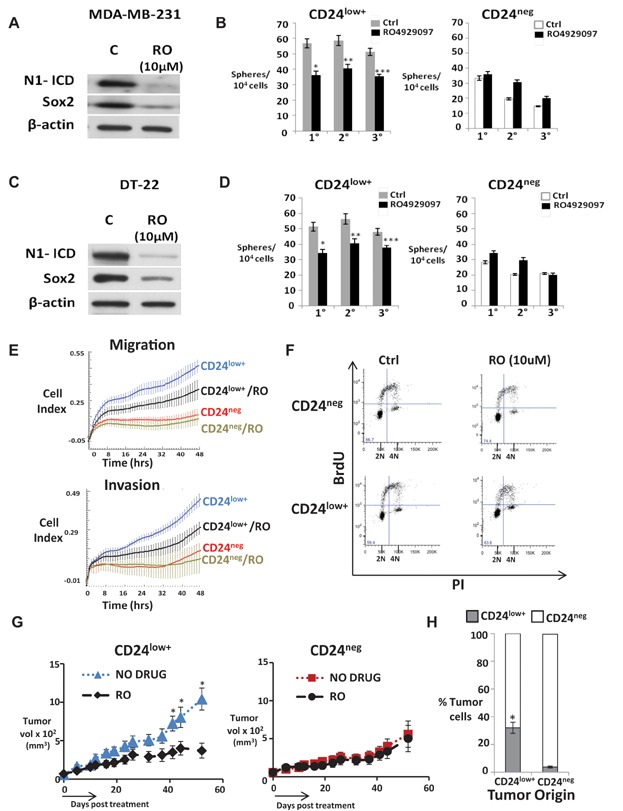
Gamma-secretase inhibitors target the CD44^+^CD24^low+^ but not the CD44^+^CD24^neg^ population CD44^+^CD24^low+^ (CD24^low+^) and CD44^+^CD24^neg^ (CD24^neg^) cells sorted from MDA-MB-231 (A and B) and DT22 (C and D) ± RO4929097. **A,C.** Cleaved Notch1 and Sox2 levels before (C) and after 24 h of 10 µM RO4929097 in CD24^low+^ cells.**B,D.** Serial mammospheres from indicated sorted cell populations ±10 µM RO4929097 (mean ± SEM, Student's *t*-test). The *p* values in panel B: *p = 0.00002; **p = 0.0018; ***p = 0.004; panel D: *p = 0.00004; **p = 0.003; ***p = 0.0015. The GSI, DAPT, showed similar effects on sorted MDA-MB-231, DT-22 and DT-25 see Supporting Information Fig S6A–F.**E.** Migration and matrigel invasion of sorted populations from MDA-MB-231 ± 10 µM RO4929097 (RO) generated by xCELLigence real time cell analysis, graphed as mean ± SD. CD24^low+^ cells show significantly greater migration and invasion compared to CD24^neg^ cells (*p* = 0.0003 at *T* = 48 h). RO significantly inhibited migration (*p* = 0.0002 at *T* = 48hrs) and invasion (*p* = 0.0002 at *T* = 48 h) of CD24^low+^ cells. CD24^neg^ cells were unaffected (see also data for DT-22 in Supporting Information Fig S6G).**F.** Cell cycle profiles of sorted DT-22 populations after 48 h ±10 µM RO were unchanged. Cell cycle profiles and proliferation curves of sorted populations were similar with and without RO treatment in MDA-MB-231 and DT-22 (see Supporting Information Fig S6H–J).**G.** Volume of DT-22 xenografts arising from CD24^low+^ or CD24^neg^ ±14-day RO4929097 treatment as described, graphed as mean ± SEM, Student's *t*-test. *p = 0.005 (Day 40); *p = 0.008 (Day 48); *p = 0.004 (Day 55).**H.** Tumours arising from sorted CD24^low+^ or CD24^neg^ DT-22 cells (*n* = 18 each group) were excised at mean 1.2 cm, dissociated into single cell suspensions and stained for surface CD44 and CD24 expression. All cells were CD44^+^. Mean CD24 expression in each tumour group is shown as a mean % of total ± SEM (*p = 0.0003, Student's *t*-test).

Similarly, N1-ICD overexpression in MDA-MB-231 increased both *SOX2* expression by qPCR and N1-ICD-binding to the *SOX2* promoter on ChIP assays (Fig 5C–E). MDA-MB-231-N1-ICD showed increased frequencies of mammosphere forming cells, CD44^+^CD24^low+^ and ALDH1^+^ cells. Notably all of these were reduced by *SOX2* knockdown (Fig 5F–H). MDA-MB-231 cell cycle profiles (Fig 5I) and proliferation curves over 12 days (Fig. 5J) were unchanged by either N1-ICD overexpression or *SOX2* knockdown. Thus, the effects of N1-ICD overexpression and *SOX2* knockdown on sphere formation were not merely attributable to effects on cell proliferation. These data suggest that Notch1 critically upregulates *SOX2*, to promote CD44^+^CD24^low+^ cell self-renewal (see also Fig 6F).

### Differential sensitivity to γ-secretase inhibitor in T-ISC populations

Notch pathway activation in T-ISC has prompted clinical trials of gamma-secretase inhibitor (GSI) drugs for various cancers (Wang et al, 2009). The GSI, RO4929097 a novel, well-tolerated drug that is in clinical trials, significantly reduced N1-ICD in CD44^+^CD24^low+^ from both MDA-MB-231 and DT22, and reduced Sox2 (Fig 6A and C). Furthermore, RO4929097 reduced sphere formation from CD44^+^CD24^low+^cells by about 50% over serial passages. Although the percent of sphere forming cells was lower in sorted CD44^+^CD24^neg^ and declined with successive passage, it was not affected by RO4929097 (Fig. 6B and D). Similar effects were observed in sorted populations from MDA-MB-231, DT-22 and DT-25 with another GSI (DAPT; see Supporting Information Fig S6A–E). Notably, colony formation by CD44^+^CD24^low+^cells was also attenuated by DAPT, but unaffected by this drug in CD44^+^CD24^neg^ cells (shown for MDA-MB-231, Supporting Information Fig S6F).

Since CD44^+^CD24^low+^ MDA-MB-231 cells generated metastatic tumours, while none emerged from CD44^+^CD24^neg^ cell injections, we investigated the importance of Notch activation in the CD44^+^CD24^low+^ population to cell migration and invasion. Assays of sorted populations using xCELLigence real time cell analysis showed significantly greater transwell migration and matrigel invasion by CD44^+^CD24^low+^ cells compared to CD44^+^CD24^neg^ cells in both DT-22 and MDA-MB-231 cells. Notably, RO4929097 significantly inhibited migration and invasion of CD44^+^CD24^low+^ cells while CD44^+^CD24^neg^ cells were unaffected (data for MDA-MB-231, Fig 6E and for DT22, Supporting Information Fig S6G).

To ensure that effects of RO4929097 on sphere formation did not result from growth or cell cycle arrest, cell cycle profiles were assayed after 48 h of drug and drug effects on cell numbers over time were assayed over 12 days in culture. Cell cycle and proliferation curves of sorted populations were similar with and without RO4929097 treatment in both DT-22 and MDA-MB-231 (Fig 6F and Supporting Information Fig S6H–J).

Differences in RO4929097 response between CD24 low and negative cells were further investigated *in vivo* with DT-22. RO4929097 treatment, initiated when tumours reached 70 mm^3^, significantly inhibited growth of CD44^+^CD24^low+^-derived xenografts but not those arising from CD44^+^CD24^neg^ cells (Fig 6G). Xenografts generated from either CD44^+^CD24^neg^ or CD44^+^CD24^low+^ sorted populations were excised, dissociated and human cells analysed for CD44 and CD24. Tumours derived from CD44^+^CD24^low+^cells (*n* = 18) were comprised of both cell phenotypes with a mean of 32 ± 4% CD24^low+^ cells and a majority CD24^neg^ cells (as observed in spheres seeded from single CD44^+^CD24^low+^cells, Fig 2F). Notably, while spheres generated from single CD44^+^CD24^neg^ cells contained only cells of like phenotype (Fig 2F), all of 18 CD44^+^CD24^neg^ cell-derived tumours showed a minor CD24^low+^component (mean 3.7 ± 0.7% cells, Fig 6H). It is noteworthy that the purity of the sorted CD24^neg^ cells was only 96% at innoculation, thus the 4% CD24^low+^ cells contaminating the sorted CD44^+^CD24^neg^ may have generated CD24^low+^ cells in the CD44^+^CD24^neg^ tumours.

### CD44^+^CD24^low+^ subpopulations exhibit heterogeneity with regard to GSI sensitivity

**Figure 7:**
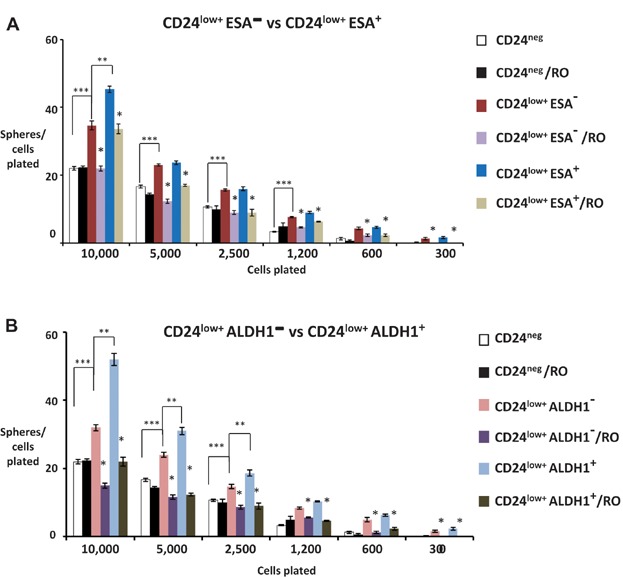
Heterogeneity within CD44^+^CD24^low+^ subpopulations with regard to GSI sensitivity MDA-MB-231 cells were flow sorted into either CD44^+^CD24^neg^, CD44^+^ CD24^low+^ ESA− and CD44^+^CD24^low+^ ESA^+^ subpopulations and analysed for frequency of sphere forming cells by limiting dilution assay ±10 µM RO4929097 (RO). Mean of spheres/cells plated are plotted from triplicate data from each of two independent experiments. The *p* values for 10,000 cells: ***p = 0.01; **p = 0.03; *p = 0.01 and 0.03; for 5000 cells: *p = 0.001 and 0.007; for 2500 cells: *p = 0.001 and 0.007; for 1250 cells: *p = 0.003 and 0.006; for 600 cells: *p = 0.001 and 0.007; for 300 cells: *p = 0.02 and 0.007 (Student's *t*-test).MDA-MB-231 cells were flow sorted into either CD44^+^CD24^neg^, CD44^+^CD24^low+^ALDH1^−^ and CD44^+^CD24^low+^ALDH1^+^ subpopulations and analysed for frequency of sphere forming cells by limiting dilution assay ±10 µM RO. Mean spheres/cells plated are plotted from triplicate data from each of two independent experiments. The *p* values for 10,000 cells: ***p = 0.01; **p = 0.01; *p = 0.001 and 0.01; for 5000 cells: ***p = 0.01; **p = 0.05; *p = 0.008 and 0.001; for 2500 cells: ***p = 0.01; **p = 0.02; *p = 0.001 and 0.003; for 1250 cells: *p = 0.047 and 0.00045; for 600 cells: *p = 0.005 and 0.00045; for 300 cells: *p = 0.007 and 0.0002 (Student's *t*-test). Cells used in (A) and (B) above were sorted simultaneously.

Heterogeneity in the stem like populations may prove to be clinically important because it may account for differences in therapeutic drug responsiveness. We had observed ALDH1^+^ and ESA^+^ cells only in the low-level CD24 positive cell fraction of TNBC lines and DTs (Fig 1). To further evaluate the heterogeneity within the CD44^+^CD24^low+^cells, these were flow sorted into ESA^+^ and ESA^−^ and ALDH1^+^ and ALDH1^−^ subsets and seeded into sphere assays with or without the GSI RO4929097. These assays were carried out in limiting dilutions, seeding sorted CD44^+^CD24^neg^ as controls, along with, CD44^+^CD24^low+^ESA^−^ or CD44^+^CD24^low+^ESA^+^ cells and CD44^+^CD24^low+^ALDH1^−^ or CD44^+^CD24^low+^ALDH1^+^ cells (10,000, 5000, 2500, 1200, 600, 300 cells). Mean sphere numbers are presented as Fig 7A and B for clarity, but numbers can be compared as they were seeded simultaneously. A modest increase in sphere formation was observed in CD44^+^CD24^low+^ESA^+^ compared to CD44^+^CD24^low+^ESA^−^ cells with higher numbers of cells plated (Fig 7A). A more significant increase in sphere formation was consistently observed in CD44^+^CD24^low+^ALDH1^+^ compared to CD44^+^CD24^low+^ALDH1^−^ cells at 10,000, 5000 and 2500 cells plated (Fig 7B). Interestingly, while RO4929097 attenuated sphere formation from all cell subsets, the ALDH1^+^ subset of CD44^+^CD24^low+^ cells appear to have the greatest spherogenicity and the greatest sensitivity to GSI.

### N1-ICD overexpression mediates infrequent conversion of CD44^+^CD24^neg^ to CD44^+^CD24^low+^

Findings to date had revealed that CD44^+^CD24^low+^ show greater sphere forming ability, more frequent tumour initiating cells (DT-22), and are driven by N1-ICD. Furthermore, kinetic data in Fig 2 and Supporting Information Fig S3 support a model in which they generate CD24 negative progeny via a largely asymmetric pattern of cell division. In contrast, in 2D and sphere culture, CD44^+^CD24^neg^ cells appear to be lineage restricted, producing only CD24 negative progeny. Notably, DT-22 tumours generated *in vivo* from the CD24 negative population were found to contain rare CD44^+^CD24^low+^ cells (<4%). While these could reflect a minor contaminating population in the initial cells injected, the possibility of a niche effect mediating conversion of the CD24 negative cells to low positive was raised.

**Figure 8:**
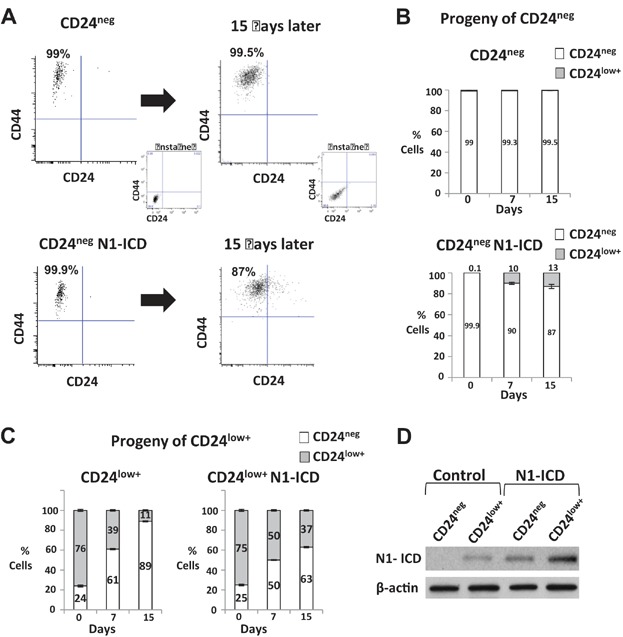
CD44^+^CD24^neg^ cells convert to CD44^+^CD24^low+^ upon Notch1 activation **A–D.** N1-ICD was expressed MDA-MB-231 and N1-ICD-CD44^+^CD24^neg^ (A,B,D) and N1-ICD-CD44^+^CD24^low+^ (C,D) populations were sorted and compared with their counterparts in vector control MDA-MB-231 cells. Progeny of each were counted, and the proportion of cells expressing CD24 analysed by flow cytometry over a period of 15 days is graphed as mean ± SEM. Note that cell number increase over time was similar with and without N1-ICD expression (Fig 5J). NI-ICD expression is shown by western blot in controls and in N1-ICD-CD44^+^CD24^neg^ and N1-ICD-CD44^+^CD24^low+^ (D).

We thus tested if N1-ICD overexpression could revert the phenotype of CD24 negative to positive. N1-ICD overexpressing MDA-MB-231 were sorted into N1-ICD-CD44^+^CD24^neg^ and N1-ICD-CD44^+^CD24^low+^ populations and compared to their vector control counterparts. Empty vector control CD44^+^CD24^neg^ cells proliferate as in Fig 2B and yield only like progeny over 15 days. In contrast, N1-ICD-CD44^+^CD24^neg^ cells had acquired the ability to generate CD24^low+^ cells and yielded a small proportion of these cells which increased from 0 to 13% over 15 days in culture (Fig 8A and B). Furthermore, the proportion of CD24^low+^ cells in the progeny of N1-ICD-CD44^+^CD24^low+^ was significantly higher than in their respective vector controls over this period (Fig 8C). Expression of N1-ICD was documented by western blot in both CD24^neg^ and CD24^low+^ sorted populations transduced with N1-ICD (Fig 8D).

## DISCUSSION

While many solid tumours appear to be driven by T-ISCs, emerging data suggest that phenotypically distinct subsets exist within T-ISC populations (Meyer et al, 2010; Patel et al, 2012; Visvader & Lindeman, 2012) and thus targeting of this population therapeutically may prove more challenging than previously appreciated. Earlier work showed primary human CD44^+^CD24^neg/low^ESA^+^ breast cancer cells were enriched for T-ISC, with <1/200 generating tumours in immune compromised hosts (Al Hajj et al, 2003). Fillmore et al. showed CD44^+^CD24^neg/low^ESA^+^ in both ER positive and ER negative breast cancer cell lines were enriched in stem cell properties and T-ISC, while the strongly surface CD24 positive CD44^+^ cells such as we observe in ER positive cancer lines were not (Fillmore & Kuperwasser, 2008). Aldefluor activity also marks stem-like properties and its combined enrichment together with surface CD44^+^CD24^neg/low^ yields a highly tumourigenic population (Ginestier et al, 2007). The CD44^+^CD24^neg/low^ primary human breast cancer population is enriched by taxane chemotherapy (Li et al, 2008), and exhibits distinct gene expression profiles, prognostic of poor patient outcomes (Creighton et al, 2009; Liu et al, 2007; Shipitsin et al, 2007; Tsunoda et al, 2011).

Here we show that cells with low level surface CD24 expression (CD24^low+^) and CD24 negative (CD24^neg^) cells comprise distinct phenotypes in the T-ISC-enriched CD44^+^CD24^neg/low^ population of TNBC lines and primary dissociated tumour cultures. The CD44^+^ population with low surface CD24 positivity (CD44^+^CD24^low+^) represents 11–23% of CD44^+^ cells, has greater sphere forming potential, increased clonogenicity and appears to give rise to CD24^neg^ progeny. CD44^+^CD24^neg^ cells show fewer sphere-generating cells (a proxy for stem cell function *in vitro*) that decline with serial passage, and generate only CD44^+^CD24^neg^ progeny in 2D and 3D culture. ESA^+^ and ALDH1^+^ cells were only seen in the CD44^+^CD24^low+^ population, revealing further heterogeneity within this phenotype. Both populations generate orthotopic xenografts, but the proportion of T-ISC in the CD44^+^CD24^low+^ population is higher (1 in 72 T-ISC frequency in CD44^+^CD24^low+^ compared to 1 in 44,936 for CD44^+^CD24^neg^ in DT-22). Notably, CD44^+^CD24^low+^ cells preferentially express ES genes and metastatic gene signatures, show greater motility and invasion, and in the MDA-MB-231 model, yield tumours that metastasize while CD44^+^CD24^neg^ did not. The CD24^neg^ subpopulation within CD44^+^CD24^neg/low^ cells showed no Notch1 activation and was GSI insensitive both in 3D (spheres and soft agar colonies) and in xenografts, highlighting important therapeutic implications of heterogeneity in cancer stem cell populations.

The difficulty of isolating sufficient T-ISCs from primary cancers to permit their molecular characterization has hampered efforts to define pathways critical for T-ISC self-renewal, therapy resistance and metastasis. The present study used not only TNBC lines, but also confirmed findings in human breast cancer cultures at early passage. Early passage patient tumour cultures—while an imperfect substitute for primary tumour analysis—permitted molecular assays in T-ISC subsets requiring large cell numbers that are not feasible in primary cancers. It is worth noting that not all TNBC lines and DTs assayed exhibit the dominant CD44/CD24 marker pattern evaluated herein. Present work addressed only the CD44^+^CD24^neg/low^ subset of TNBC. Further investigation is required to determine its relevance to T-ISC in other breast cancer types.

In all three models assayed, CD44^+^CD24^low+^ cells gave rise to both CD44^+^CD24^low+^ and CD44^+^CD24^neg^ phenotypes in 2D culture with asymmetric growth kinetics. Spheres seeded by single CD44^+^CD24^low+^ cells comprised progeny – both phenotypes, while spheres generated by single CD44^+^CD24^neg^ cells yielded only CD44^+^CD24^neg^, suggesting that CD24^neg^ arise from CD24^low^ and are restricted to produce only like progeny. Notably, while tumours generated from sorted CD44^+^CD24^low+^ DT-22 cells comprised both phenotypes, tumours arising from sorted CD44^+^CD24^neg^ cells were almost entirely CD44^+^CD24^neg^. The 3–4% CD44^+^CD24^low+^ cells present in all of 18 orthotopic tumours generated from CD44^+^CD24^neg^-enriched cells may reflect the lack of purity in the initial sorted cells injected (4% CD24^low^ present in the CD24^neg^ enriched population) or could represent phenotypic conversion within a cancer population from a lower to a greater self-renewal potential (discussed further below). These reproducible patterns seen not only in MDA-MB-231, but also in two independent primary TNBC tumour-derived cultures, support a lineage relationship rather than random T-ISC heterogeneity.

The genetic plasticity and aberrant differentiation within cancer cell populations would permit clonal evolution within T-ISC populations, but do not preclude the existence of abortive developmental hierarchies that could contribute to heterogeneity. While other explanations are possible, our data are compatible with a hierarchy such as what has been observed in human leukaemia stem cell subsets (Goardon et al, 2011), colon (Bu et al, 2013) and liver cancer T-ISC (Lee et al, [Bibr b37]). Further evidence for the existence of stem-like cancer cells giving rise to clonally related cells with reduced self-renewal capacity was recently provided by lineage tracing in squamous skin papilloma (Driessens et al, 2012). Present data support the notion that precursor–progeny relationships can exist in T-ISC populations, yielding subsets that differ in self-renewal and, potentially, in the ability to establish metastasis (Dalerba et al, 2007).

CD24 may be a marker of metastatic potential. Its expression is increased in breast and bladder cancer metastasis compared to primaries and confers poor prognosis (Athanassiadou et al, 2009; Bircan et al, 2006; Overdevest et al, 2011; Shipitsin et al, 2007) and CD24 knockdown abrogates metastasis in bladder cancer (Overdevest et al, 2011). Present data link low level surface CD24 to metastatic potential in TNBC. In the MDA-MB-231, as few as 100 CD44^+^CD24^low+^ cells yielded orthotopic tumours that generated multi-organ metastasis and lung micrometastasis, while tumours from up to a half million CD44^+^CD24^neg^ cells failed to metastasize. Moreover, CD44^+^CD24^low+^ cells in both MDA-MB-231 and DT-22 showed greater motility and invasion, and preferential expression of gene profiles observed in breast cancers metastatic to lung (Minn et al, 2005) or brain (Bos et al, 2009) and that characterize bone metastasis (Kang et al, 2003). A T-ISC hierarchy with progressive loss of both CD24 and metastatic potential was also observed in a hepatocellular cancer, in which a highly metastatic CD24^+^ T-ISC population gave rise to CD24^neg^ cells that failed to metastasize (Lee et al, [Bibr b37]). While present work provides novel evidence for a putative metastatic hierarchy within breast cancer T-ISC in the MDA-MB-231 line, this remains to be confirmed in additional models since DT-22 does not form tumours that metastasize.

Present data provide not only an example of heterogeneity in T-ISC of TNBC, but also in molecular pathways and susceptibility to therapeutic targeting. Notch family members play complex and critical roles in fate determination in mammogenesis (Bouras et al, 2008; Raouf et al, 2008; Sale et al, 2013) and are implicated in breast cancer stem cell self-renewal (Harrison et al, 2010; Sansone et al, 2007; Wang et al, 2009). *NOTCH1* and *NOTCH4* pro-viral integration sites mediate mammary tumour formation (Dievart et al, 1999; Gallahan & Callahan, 1987), Notch4 is upregulated in T-ISC in primary non-invasive (Farnie et al, 2007) and invasive ER positive breast cancers (Harrison et al, 2010) and Notch1 overexpression in breast cancer correlates with worse prognosis (Reedijk et al, 2005; Stylianou et al, 2006).

Here we observed higher *NOTCH 1*, *2* and *4* gene expression in CD44^+^CD24^low+^ cells in MDA-MB-231 and higher levels of N1-ICD, N2-ICD and N4-ICD in both DT-22 and MDA-MB-231 models. Expression profiling showed that Notch driven genes, ES signature genes (Assou et al, 2007), genes targeted by Nanog, Oct4 and Sox2 (NOS targets) and a subset of these with transcriptional function (NOS-TFs; Boyer et al, 2005) were preferentially expressed by CD24^low+^ compared to CD24^neg^ cells. Moreover, Sox2 and Nanog proteins were higher in the Notch-activated CD24^low+^ cells, leading us to investigate the link between Notch and Sox2. Sox2 is a driver of ES self-renewal and may play a role in human cancers (Leis et al, 2011; Nakatsugawa et al, 2011; Sarkar & Hochedlinger, 2013; Xiang et al, 2011). Notch inhibition reduced Sox2, sphere and colony formation, and *in vivo* tumour growth exclusively in the CD44^+^CD24^low+^ population, without affecting global cell proliferation. Of the four different Notch genes, only N1-ICD overexpression significantly induced *SOX2* in our mammary cell models, and N1-ICD bound the *SOX2* promoter in both. Moreover, N1-ICD overexpressing MDA-MB-231 showed a Sox2-dependent increase in the proportions of ALDH1^+^ and of CD44^+^CD24^low+^ cells, and in sphere formation, suggesting that Notch1 critically activates *SOX2* to drive T-ISC self-renewal in these ER negative breast cancer models.

Heterogeneity in driving pathways and in the phenotypes of T-ISC has become increasingly apparent (Patel et al, 2012; Schober & Fuchs, 2011; Visvader & Lindeman, 2012). T-ISC of different surface phenotypes and metastatic ability have been shown to co-exist in pancreatic (Hermann et al, 2007), colon (Pang et al, 2010) and liver cancer models (Lee et al, [Bibr b37]; Yang et al, 2008). Recent work by Kim et al showed that tumour initiating cells in breast cancers of either basal or luminal phenotypes co-exist, with the latter generating more invasive tumours. They also provide evidence that basal-like cancer cells with stem cell traits may give rise to progeny cells with luminal phenotype but not vice versa and cells with luminal markers can initiate tumours (Kim et al, 2012).

The present study provides evidence for two phenotypically distinct populations within the TNBC CD44^+^CD24^neg/low^, in which CD44^+^CD24^neg^ arise from a CD44^+^CD24^low+^ precursor population. We also show evidence for phenotypic heterogeneity within the CD44^+^CD24^low+^ population which comprises subsets of ESA^+^ and ESA^−^ and ALDH1^+^ and ALDH1^−^ cells with different levels of self-renewal as evidenced by limiting dilution sphere assays. Whether precursor–progeny relationships exist within the ESA and ALDH1-based subgroups is yet unknown. ALDH1 activity is very infrequent in CD44^+^CD24^neg/low^ breast cancer cells (Charafe-Jauffret et al, 2009; Croker et al, 2008; Ginestier et al, 2007), and populations enriched for both aldefluor activity and CD44^+^CD24^neg/low^ show a high T-ISC frequency (Ginestier et al, 2007). It is noteworthy that our ALDH1^+^ CD44^+^CD24^low+^ cells showed the greatest abundance of sphere forming cells and also the greatest responsiveness to Notch pathway inhibition.

Several groups have presented evidence for potential phenotypic conversion within marker-defined populations (Meyer et al, 2009). In *tert*-immortalized and oncogene transformed HMEC models, CD44 negative cells have been shown to generate CD44 positive progeny suggesting that more “differentiated cells” can revert to a more primordial stem-like precursor (Chaffer et al, 2011). Different subsets within ER negative breast cancer lines sorted by CD44, CD24 and ESA status appear to undergo transitions compatible with a model of stochastic phenotypic conversion (Gupta et al, 2011). Here we show that N1-ICD overexpression not only increased CD24^low+^ cells, suggesting an increase in self-renewal/symmetric division, but also led to a low rate of conversion of CD44^+^CD24^neg^ cell to CD44^+^CD24^low+^, an event we have not observed spontaneously in culture over several years. Interestingly, xenografts generated from sorted CD44^+^CD24^neg^ DT-22 cells consistently contained 2–8% CD44^+^CD24^low^ cells in all of 18 tumours, raising the possibility of a niche-induced inter-conversion *in vivo*. Taken together, these observations raise the intriguing possibility that the tumour microenvironment may regulate the phenotypic heterogeneity present within tumour initiating stem cells as observed in other systems (Lu et al, 2013).

An important implication of heterogeneity within malignant stem cell populations is that more primitive stem cells may have not only a greater metastatic propensity, they may also differ from their bulk progeny and escape therapy to re-populate. The importance of the Notch pathway to mammogenesis and mammary cancer has led to the clinical development of Notch inhibitors (Morgan et al, 2013), several of which are in clinical trials. Notch inhibitors have been shown to augment pre-clinical efficacy of estrogen receptor blockade (Rizzo et al, 2008), trastuzumab (Osipo et al, 2008) and radiation (Phillips et al, 2006), and enhance chemotherapy response through depletion of stem cells in preclinical models (Qiu et al, 2013) and clinical trials (Schott et al, 2013). They may also have particular efficacy in TNBC (Clementz et al, 2011; O'Toole et al, 2013). Loss of experimental brain metastasis following Notch1 knockdown in xenograft models lends support for Notch-targeted therapies (McGowan et al, 2011).

T-ISC heterogeneity may limit the efficacy of therapies that oppose Notch signalling. The two TNBC T-ISC populations characterized herein, differed notably in their responses to GSI. RO4929097, a GSI in clinical trials for cancer, had no effect on tumours derived from CD44^+^CD24^neg^ cells, which comprise a majority of the population in TNBC lines. Only CD44^+^CD24^low+^-generated tumours responded to RO4929097. Even in the N1-ICD-expressing CD44^+^CD24^low+^ population, sphere formation and tumour growth were not fully abrogated by GSI treatment. This could reflect incomplete Notch inhibition or the presence of a Notch-independent CD44^+^CD24^low+^ subpopulation. While Notch pathway inhibitors hold promise for preferentially targeting the more malignant T-ISC, Notch1/Sox2 dependence may be restricted within T-ISC subsets. The presence of functionally discrete T-ISC subpopulations may also limit tumour responses to other targeted therapies. Present findings support further phenotypic and molecular characterization of distinct T-ISC subpopulations, since they may illuminate limitations of current therapy and open new avenues for more effective cancer treatment.

## MATERIALS AND METHODS

### Cell lines and primary dissociated tumour cell cultures

MDA-MB-231-luc were cultured as described in Minn et al (2005). Primary dissociated breast tumour (DT) cultures were isolated by density gradient centrifugation after mechanical dissociation of primary triple negative breast cancers and propagated as described (Bayliss et al, 2007). DT-22 and DT-25 are from basal subtype tumours as defined by microarray profiling, DT-28 shows a basal/epithelial phenotype, DT-13 is in the HER2 group, and DT-16 has a luminal B expression profile. DT-21 and DT-23 have expression profiles of cancer associated fibroblasts. MCF-10A is a spontaneously immortalized non-tumourigenic human mammary epithelial line. All DTs were grown in IMEM with 10% FBS, used between population doubling 3–30 and passaged 1:2. Established lines were grown in DMEM with 10% FBS.

### Flow cytometric assays

Antibodies specific to human cell surface markers: phycoerythrin (PE)-conjugated anti-human CD24 mAb, allophycocyanin (APC)-conjugated anti-human CD44 mAb (BD Pharmingen, CA) and FITC-conjugated mAb to human ESA (Biomeda) were used as in (Fillmore & Kuperwasser, 2008). Flow cytometry for ALDH1 staining as in (Ginestier et al, 2007). The gating strategy for isolating CD44^+^CD24^low+^ ESA^−^, CD44^+^CD24^low+^ ESA^+^, CD44^+^CD24^low+^ALDH1^−^ and CD44^+^CD24^low+^ALDH1^+^ was as shown in Fig 1F and G. Cell cycle distribution was assayed by bromodeoxyuridine (BrdU) pulse labelling and flow cytometry as described in (Larrea et al, 2009).

### Flow sorting of CD44^+^CD24^neg^ and CD44^+^CD24^low+^ cells

Cells were labelled with APC-conjugated-CD44 and PE-conjugated CD24 mAbs for 25 min at 22°C. Magnetic separation of CD24^low+^ and CD24^neg^ cells was performed on anti-PE magnetic beads (PE-selection kit, Stem Cell Tech) four times to obtain >90% purity of CD24^low+^ cells. CD24^neg^ cells were then flow sorted to >98% purity (FACSAriaII BD Biosciences).

### Sphere formation assay and growth in soft agar

Mammosphere assays were as described in Dontu et al (2003), using 10,000 cells/6-well plates. Spheres >75 µm were counted at 7–10 days. For serial sphere assays, spheres were counted, collected by centrifugation, dissociated with trypsin, re-seeded and evaluated as above. Soft agar colonies were seeded at 10,000 cells per ml of soft agar, stained with 0.1% crystal violet and >75 µm counted at 3–4 weeks.

### Drug effects on proliferation

Flow sorted CD44^+^CD24^neg^ and CD44^+^CD24^low+^ populations were treated with or without GSI drugs DAPT 5 (µM) and RO4920927 (10 µM) for 48 h prior to flow cytometry for cell cycle distribution as described (Larrea et al, 2009). CD44^+^CD24^neg^ and CD44^+^CD24^low+^ cells (100,000 cells/well) were seeded with or without RO4920927 (10 µM) and viable cells were counted at 4, 8 and 12 days with drugs renewed every 4 days.

### Western analysis

Westerns were as described (Ginestier et al, 2007), using antibodies to cleaved Notch1, Sox2, Nanog and β-actin (Cell Signaling Tech, MA). Antibodies to Notch2-ICD, Notch3-ICD and Notch4-ICD were from Abcam.

### Chromatin immunoprecipitation assay

ChIP assays were as described (Assou et al, 2007) and used anti-cleaved Notch1 antibody or control IgG to precipitated DNA −770 to −616 from the human *SOX2* promoter start site. See human and mouse PCR primers in Supporting Informaton Methods.

### *In vivo* tumourigenicity

Sorted cells from MDA-MB-231-luc (500,000, 100,000, 10,000, 1000 or 100) were injected in 0.05 ml matrigel into one inguinal mammary fat pad of 4- to 6-week-old Balb/C nude mice (Charles River) and tumours measured twice weekly. Tumours from the 500,000 cell group were excised at 1 cm diameter and followed for metastasis. All others were excised per Animal Care and Use Committee or at 100 days if no tumour arose.

DT-22 cells were luciferase tagged and 100,000, 10,000 or 100 sorted cells were xenografted into nude mice (*n* = 10–12/group). When 100,000 cells formed palpable tumours (mean 70 mm^3^), animals were treated with either vehicle or RO4929097 (Selleck Chemicals) 30 mg/kg/day for 14 days as in (Luistro et al, 2009; *n* = 8/group) and measured twice weekly. Bioluminescence images were acquired by IVIS as in (Minn et al, 2005) with Living Image 3.0 software (Xenogen Caliper Life Sciences). Tumours were excised at 1.2 cm diameter and single-cell suspensions prepared as in (Charafe-Jauffret et al, 2009), CD44 and CD24 stained and analysed by flow cytometry. Following primary tumour excision, none of the DT-22-derived tumours yielded metastasis over 9 months of follow-up.

**The paper explained**

**PROBLEM:**

Most cancer patient death is due to progression of visceral metastasis resistant to chemo and radiation therapies. Breast and other common cancers appear to arise from stem-like cells that self-renew and give rise to progeny with less robust self-renewal. These tumour-initiating stem cells or cancer stem cells (T-ISC/CSCs) express discrete surface markers, can generate tumours from as few as 10–100 cells and may have greater ability to form metastasis and resist treatment than the bulk tumour population in experimental models. Hence, drugs that preferentially target the CSCs may more effectively eradicate cancers. Defining how the CSC differ from bulk tumour cells and whether unique pathways drive CSC is of paramount importance for development CSC-targeted therapy. The present study was undertaken to test if the CD44^+^CD24^neg/low^ breast cancer stem cell population may contain heterogeneous subsets of differing malignant potential that could subvert effective therapeutic targeting.

**RESULTS:**

In immortal cancer-derived lines and early cultures from primary breast cancers two different, potentially related subsets were identified within the CD44^+^CD24^neg/low^ CSC population of a deadly form of breast cancer (triple-negative breast cancer or TNBC): a minor CD44^+^CD24^low+^ subpopulation generates CD44^+^CD24^neg^ progeny with reduced self-renewal and different functional and molecular characteristics. The CD44^+^CD24^low+^ population showed a greater proportion of cells that can initiate tumours in immunocompromised mice, preferentially expressed genes that predict for metastasis in breast cancer patients and gave rise to metastasis. The Notch pathway, which is known to drive self-renewal of embryonic stem cell and certain cancer stem cells, was strongly activated in CD44^+^CD24^low+^ cells but not in the CD44^+^CD24^neg^ cells. Notably, Notch pathway inhibition by gamma-secretase inhibitor (GSI) drugs inhibited growth of CD44^+^CD24^low+^-generated tumours but those from CD44^+^CD24^neg^ were completely drug resistant.

**IMPACT:**

Treating cancers with stem cell targeted therapies may be more complicated than we thought. While GSI drugs hold promise for targeting breast CSCs, heterogeneity within the T-ISC subpopulation could limit their therapeutic efficacy. CSC subpopulations within a tumour may be driven by different molecular pathways and thus differ in their ability to respond to drugs. These findings support further phenotypic and molecular characterization of distinct T-ISC subpopulations, since they may illuminate limitations of current therapy and open new avenues for more effective cancer treatment.

### Microarray data acquisition, processing and gene set enrichment analysis

RNA was isolated with miRNeasy kit (Qiagen) and quantified by Nanodrop 8000 Spectrophotometer (Thermo Scientific, Wilmington) and quality verified by RNA 6000 Nano kit (Agilent, Santa Clara, CA) on a Bioanalyzer 2100 and expression analysis used the Illumina platform (see Supporting Information Methods). Expression data of gene sets from previously described lung, bone and brain metastasis gene signatures were obtained from NCBI Gene Expression Omnibus (accession numbers GSE2603 and GSE12237) and embryonic stem cell transcriptional programs were obtained from ArrayExpress (http://www.ebi.ac.uk/arrayexpress) under the accession designation E-WMIT-5 (Ben-Porath et al, 2008; Bos et al, 2009; Kang et al, 2003; Minn et al, 2005). In the case of the NOTCH targets, this gene set was defined by GSI washout of a metastatic MDA-MB-231 variant (see Supporting Information Methods).

Microarray data processing and analysis were performed as described (Minn et al, 2005) using R language and environment for statistical computing version 2.13 and Bioconductor version 2.8. gene set analysis (GSA) was performed using the GSA R package version 1.03. Gene sets tested were obtained from the original publications (Ben-Porath et al, 2008; Bos et al, 2009; Charafe-Jauffret et al, 2009; Creighton et al, 2009; Kang et al, 2003; Liu et al, 2007; Minn et al, 2005). In all cases, a two-class paired comparison between CD24^neg^ and CD24^low^ cells was applied using the maxmean statistic and re-standardization based on all microarray data set genes. Gene sets showing positive or negative enrichment were significant if false discovery rates and nominal p-values were <0.05 using 1000 permutations. See Supporting Information Methods for details.

### Automated migration and invasion assays

Automated transwell migration and invasion assays used the Real-Time Cell Analysis (RTCA) system from xCELLigence. For both assays, 20,000 cells were seeded onto a semipermeable membrane without matrigel (migration) or with matrigel (invasion) in the upper chamber in serum-free medium, with 10% FBS added in the bottom chamber. Cell movement across the membrane was measured over 48 h.

### N1-ICD overexpression and siRNA transfection

HC-11 or MDA-MB-231 were transduced with pBABEpuro-N1-ICD retrovirus. For Sox2 knockdown, scrambled controls or siRNAs against Sox2 (Santa Cruz) were transfected into MDA-MB-231 with Lipofectamine 2000.

### Statistical analysis

Data were presented as mean ± SE from at least three experiments and used two-tailed Student's *t* tests to detect differences. Comparative analysis of growth curves (http://bioinf.wehi.edu.au/software/compareCurves) was applied to tumour growth curves over a series of multiple time points using the statmod software package. TISC frequency was calculated using the L-Calc software http://www.stemcell.com/tutorials/lcsetup.exe (Stem Cell Technologies). Microarray analysis is described above and in Supporting Information Methods and used MIAME standards.

## Supplementary material

As a service to our authors and readers, this journal provides supporting information supplied by the authors. Such materials are peer reviewed and may be re-organized for online delivery, but are not copy-edited or typeset. Technical support issues arising from supporting information (other than missing files) should be addressed to the authors.

**Figure S1. Frequent observation of CD44**^**+**^**CD24**^**neg/lo**^ **surface expression in ER negative breast cancer lines and tumour cultures.** (A) ER negative breast cancer cell lines. (B) TNBC-derived dissociated tumour (DT) cultures. (C) ER positive lines and (D) equal numbers of mixed MDA-MB-231 and MCF7 cells.

**Figure S2. ESA expression and aldefluor activity are observed in subsets of CD44**^**+**^**CD24**^**low+**^ **cells.** (A) CD44 and CD24 in DT-25 at passage (P3) compared to DT-25 at passage (P9). (B) Serial mammospheres formed from sorted CD44^+^CD24^low+^ and CD44^+^CD24^neg^ from DT-25. Mean ± SEM, **p* = 0.0054. (C) Aldefluor activity (top) and flow cytometry for surface ESA (bottom) were assayed as described in MDA-MB-231 and DT-22. Respective unstained controls are shown. (D) Cells were gated for ESA^+^ (left) or ALDH1^+^ (right) and CD44/CD24 assayed.

**Figure S3. CD44**^**+**^**CD24**^**low+**^ **give rise to both CD44**^**+**^**CD24**^**low+**^ **and CD44**^**+**^**CD24**^**neg**^ **progeny while CD44**^**+**^**CD24**^**neg**^ **yield only CD44**^**+**^**CD24**^**neg**^**.** CD44^+^CD24^low+^ or CD44^+^CD24^neg^ were sorted from DT-22 and DT25 and 100,000 cells cultured. (A and D) Population growth from sorted CD44^+^CD24^low+^ and CD44^+^CD24^neg^. (B and E.) The proportion of CD44+CD24^low+^ or CD44^+^CD24^neg^ cells arising from CD44^+^CD24^low+^ cells is shown over time. (C and F) Growth curves of progeny of CD44^+^CD24^low+^ over 14 days. As for MDA-MB-231 (Fig 3A) CD44^+^CD24^neg^ generated only CD44^+^CD24^neg^ cells over 14 days (not shown here).

**Figure S4. Expression of lung, brain and bone metastasis gene expression signatures is enriched in CD44**^**+**^**CD24**^**low+**^ **subpopulations from MDA-MB-231 and DT-22.** (A–C) Expression of lung (A), brain (B) and bone (C) metastasis gene expression signatures is enriched in CD44^+^CD24^low+^ subpopulations in MDA-MB-231. Gene set analysis (GSA) compared enrichment of indicated signatures. Shown are ordered gene scores for each gene in the line plot and the average fold change in the heatmap (orange indicates high expression in CD44^+^CD24^low+^ and blue is low). Average fold gene expression changes are indicated by bar graphs. Upper left panel shows enrichment score and the *p*-value. (D and E) Expression of lung (D) and brain (E) metastasis gene expression signatures is enriched in CD44^+^CD24^low+^ subpopulations in DT-22 analysis as above. A list of the genes used for each signature and their individual gene scores are in shown in Supporting Information Table S1.

**Figure S5. Expression of embryonic stem cell transcription factors and targets is enriched in CD44**^**+**^**CD24**^**low+**^ **subpopulations.** (A–F) GSA comparing enrichment of indicated embryonic stem cell transcription factors (A, B, D and E) and genes upregulated by NOS (Nanog, Oct4 and Sox2). (C and F). The “NOS TF up” is a gene set of transcriptional regulators identified in human ES that overexpress Nanog, Oct4 or Sox2. The “NOS Targets up” gene set contains the activated genes from the ChIP-array for Nanog, Oct4 and Sox2. A list of the genes used for each signature and their individual gene scores are in shown in Supporting Information Table S1. For MDA-MB-231 (A–C) and DT-22 (D–F) the ordered scores for each gene in the line plot and the average fold change in the heatmap are shown (orange indicates high expression in CD44^+^CD24^low+^ and blue, low). Enrichment scores and *p* values are at upper left. Average fold gene expression changes are shown in bar graphs and a list of genes and their individual gene scores are in Supporting Information Table S1.

**Figure S6. GSI sensitivity is restricted to the CD44**^**+**^**CD24**^**low+**^**.** (A–E) CD44^+^CD24^low+^ (CD24^low+^) and CD44^+^CD24^neg^ (CD24^neg^) cells sorted from MDA-MB-231 (A and B), DT-22 (C and D) and DT-25 (E) were treated with or without DAPT. Effect of DAPT on cleaved Notch1 (N1-ICD) and Sox2 ± after 24 h of 5 and 10 µM DAPT in CD44^+^CD24^low+^ cells (A and C). Serial mammospheres of indicated cells from sorted populations ±5 μM DAPT (mean ± SEM, Student's *t*-test). The *p* values in panel B: **p* = 0.00001; ***p* = 0.00002; ****p* = 0.0001; panel D: **p* = 0.0045; ***p* = 0.006; ****p* = 0.0054; panel 10. (E) **p* = 0.0027; ***p* = 0.0005; ****p* = 0.007 Serial mammospheres from DAPT-treated CD24^low+^ cells were significantly reduced compared to respective untreated controls. *Statistically different by student's *t*-test from control primary spheres. Treated and untreated tertiary mammospheres from CD24^neg^ cells were reduced significantly compared to primary spheres, but both were unaffected by DAPT (B, D and E). (F) Mean soft agar colonies arising from sorted populations of MDA-MB-231 ±5 μM DAPT (mean ± SEM, *t*-test, **p* = 0.04). (G) Matrigel invasion of sorted populations from DT-22 ±10 µM RO4929097 (RO) generated by xCELLigence real time cell analysis, graphed as mean ± SEM. CD24^low+^ cells show significantly greater invasion compared to CD24^neg^ cells (***p* = 0.0055 at *T* = 12 hrs, Student's *t*-test). RO significantly attenuated invasion (**p* = 0.004 at *T* = 12 h, Student's *t*-test) of CD24^low+^ cells. CD24^neg^ cells were unaffected. (H) Cell cycle profiles of sorted populations from MDA-MB-231 after 48 h ±10 µM RO4929097. (I and J) GSI does not affect MDA-MB-231 or DT-22 cell proliferation. Proliferation curves of 100,000 cells from sorted CD44^+^CD24^low+^ or CD44^+^CD24^neg^ populations from MDA-MB-231 (I) and DT22 (J) cultured over 12 days ±10 µM RO4929097.

**Table S1.** Individual gene scores from GSA results showing enrichment of various gene signatures in sorted CD44^+^CD24^low+^ compared to CD44^+^CD24^neg^ subpopulations from MDA-MB-231 and DT-22 cells. Higher gene scores indicate increased expression while lower gene scores indicate decreased expression.
