# Supplementary material for: Triple negative breast cancer initiating cell subsets differ in functional and molecular characteristics and in γ-secretase inhibitor drug responses
Source: EMBO Mol Med. 2013 Aug 27;5(10):1502–22. doi: 10.1002/emmm.201302558 (PMC3799576; doi:10.1002/emmm.201302558)
Supplement: Supplementary file 1 [file emmm0005-1502-SD1.pdf]

EMBO Molecular Medicine

Triple negative breast cancer initiating cell subsets differ in functional and molecular characteristics and in γ-secretase inhibitor drug responses

Diana J. Azzam, Dekuang Zhao, Jun Sun, Andy J. Minn, Prathibha Ranganathan, Katherine Drews-Elger, Xiaoqing Han, Manuel Picon-Ruiz, Candace A. Gilbert, Seth A. Wander, Anthony J. Capobianco, Dorraya El-Ashry and Joyce M. Slingerland

Corresponding author: Joyce M. Slingerland, University of Miami Miller School of Medicine

Review timeline:

Submission date:	25 January 2013
Editorial Decision:	04 March 2013
Revision received:	07 June 2013
Editorial Decision:	08 July 2013
Revision received:	15 July 2013
Accepted:	16 July 2013

Transaction Report:

(Note: With the exception of the correction of typographical or spelling errors that could be a source of ambiguity, letters and reports are not edited. The original formatting of letters and referee reports may not be reflected in this compilation.)

Editor: Roberto Buccione

1st Editorial Decision 04 March 2013

Thank you for the submission of your manuscript to EMBO Molecular Medicine. We have now received reports from the three Reviewers whom we asked to evaluate your manuscript

You will see that while the Reviewers are generally supportive, critical points are raised that question the impact and conclusiveness of the results, preventing us from considering publication at this time.

Reviewer 1 would like to see how the original tumours reflect the phenotype described and feels that you should focus more on the Roche gamma-secretase inhibitor rather than on DAPT. S/he also mentions other issues that require your action.

Reviewer 2 notes that it remains unclear whether Notch ligands can induce a CD24- to CD24+ conversion. S/he also notes a number of mis-citations that need to be remedied.

Reviewer 3 acknowledges the clear finding that the Notch pathway is specifically active in defined populations on tumour cells, but points to a number of shortcomings that mostly concern the lack of clear distinction between proliferation and self-renewal and which require substantial additional work:
1) The lack of limited dilution assays to quantitate mammosphere formation impairs the ability to determine which cells actually form them. The Reviewer suggests that, at the very least, it should be determined whether TICs can be further enriched at least from the MDA-MB-231 cell line.

2) With reference to figure 4, the Reviewer notes that to establish differences in tumour initiation, limited dilution assays must be performed.
3) Considering the well-known propensity of MDA-MB-231 cells to form metastases, Reviewer 3 is surprised that no lung metastases are reported. S/he also suggests that it would be quite important to establish whether CD24- and CD24+ cells have different migration potential and whether Notch signalling has an impact on this aspect.
4) Reviewer 2 also notes that the effects of NOTCH inhibition and SOX2-knockdown on mammosphere formation should be integrated with proliferation assays to distinguish between general anti-proliferative/cytotoxic effects and specific effects on mammosphere formation.

While publication of the paper cannot be considered at this stage, we would be prepared to consider a suitably revised submission, with the understanding that the Reviewers' concerns must be fully addressed with additional experimental data where appropriate and that acceptance of the manuscript will entail a second round of review.

Please note that it is EMBO Molecular Medicine policy to allow a single round of revision only and that, therefore, acceptance or rejection of the manuscript will depend on the completeness of your responses included in the next, final version of the manuscript.

Since the required revision in this case appears to require a significant amount of time, additional work and experimentation and might be technically challenging, I would therefore understand if you chose to rather seek publication elsewhere at this stage. Should you do so, we would welcome a message to this effect.

As you know, EMBO Molecular Medicine has a "scooping protection" policy, whereby similar findings that are published by others during review or revision are not a criterion for rejection. However, I do ask you to get in touch with us after three months if you have not completed your revision, to update us on the status. Please also contact us as soon as possible if similar work is published elsewhere.

I look forward to seeing a revised form of your manuscript as soon as possible.

***** Reviewer's comments *****

Referee #1 (Remarks):

The authors have further characterized a subpopulation of triple negative breast cancer (TNBC) cell lines and patient derived cells for their ability to from spheres in vitro and to proliferate in vivo. In agreement with previous results, they show that the CD44+/CD24low fraction behaves as a bona fide tumor initiation cells population, e.g. it is able to generate both CD44+/Cd24low and CD44+/CD24- populations. One of their notable findings is the fact that the CD44+/CD24low cells appear to be expressing the activated Notch ICD and to be sensitive to Notch inhibition in vivo.

Unfortunately there are some elements missing for this story for it to be truly impactful. First, the authors should have established a better correlation between the TNBC samples being studies and the original tumors, for example, how well did the original tumor reflect the phenotypes described in terms of marker and notch activation staining (frequency, would there be a way to identify patients subsets that might be eligible for clinical trials); second, why would they insist on making a point on the metastatic potential of the CD44+/Cd24low cells when this property seems to be unique to MDA-MB-231; third, why bother trying to establish a connection between Sox-2 and Notch, without providing much stronger evidence supporting it; fourth, why not focus on the characterization of the Roche Notch inhibitor, rather than DAPT, making it a more coherent study (move the Roche data to Figure 6/7 from supplement).

More details on cell passages, media utilized should be provided; in our own experience cell passaging affects dramatically TIC phenotypes.

I would also ask the authors to refer to these two manuscripts:

Specific inhibition of Notch1 signaling enhances the antitumor efficacy of chemotherapy in triple negative breast cancer through reduction of cancer stem cells.Qiu M, Peng Q, Jiang I, Carroll C, Han G, Rymer I, Lippincott J, Zachwieja J, Gajiwala K, Kraynov E, Thibault S, Stone D, Gao Y, Sofia S, Gallo J, Li G, Yang J, Li K, Wei P. Cancer Lett. 2013 Jan 28;328(2):261-70. doi: 10.1016/j.canlet.2012.09.023. Epub 2012 Oct 3.
Characteristic genes in luminal subtype breast tumors with CD44+CD24-/low gene expression signature.Tsunoda Y, Sakamoto M, Sawada T, Sasaki A, Yamamoto G, Tachikawa T. Oncology. 2011;81(5-6):336-44. doi: 10.1159/000334690. Epub 2012 Jan 11.

Referee #2 (Comments on Novelty/Model System):

The paper reports that 2 cell populations with differing tumour-initiating capacity exist in triple negative breast cancer. Both in vitro and in vivo, these populations are organised as a cellular hierarchy with one population being the progeny of the other but not vice versa. Interestingly, the authors demonstrate that the bipotent CD24+ population generate self-renewing sphere colonies, have an Aldefluor+ population and are more efficient at producing xenograft tumours. Importantly, only the bipotent CD24+ population can spontaneously metastasise from a sub-cutaneous tumour xenograft. Finally, the authors demonstrate that this CD24+ population and its stem cell activity is dependent on Notch1 activity and the downstream transcription factor Sox2. Gamma secretase inhibitors that target notch1 activity can abrogate the tumorigenic and stem cell activities of the CD24+ population but do not affect the CD24-negative progeny.
The strengths include use of patient-derived dissociated breast tumour cells to complement the cell line work, in vivo limiting dilution transplantation, and mechanistic insight into the regulation of the stem cell-like CD24+ population.

Referee #2 (Remarks):

The paper reports that 2 cell populations with differing tumour-initiating capacity exist in triple negative breast cancer. Both in vitro and in vivo, these populations are organised as a cellular hierarchy with one population being the progeny of the other but not vice versa. Interestingly, the authors demonstrate that the bipotent CD24+ population generate self-renewing sphere colonies, have an Aldefluor+ population and are more efficient at producing xenograft tumours. Importantly, only the bipotent CD24+ population can spontaneously metastasise from a sub-cutaneous tumour xenograft. Finally, the authors demonstrate that this CD24+ population and its stem cell activity is dependent on Notch1 activity and the downstream transcription factor Sox2. Gamma secretase inhibitors that target notch1 activity can abrogate the tumorigenic and stem cell activities of the CD24+ population but do not affect the CD24-negative progeny.
The strengths include use of patient-derived dissociated breast tumour cells to complement the cell line work, in vivo limiting dilution transplantation, and mechanistic insight into the regulation of the stem cell-like CD24+ population.
Comments:
1. Meyer et al 2009 suggests Nodal/activin signalling can regulate interconversion between the CD24- and CD24+ phenotypes. The current paper reports that Notch inhibition prevents CD24+ to CD24- conversion but the outstanding question is can Activin or Notch ligands induce CD24- to CD24+ interconversion. It will be important to address this point.
2. Several times it is indicated that other Notch receptors are not involved for which we are shown little evidence and is conflicting with the literature, in particular Rizzo et al 2008, who show Notch4 is activated downstream of Notch1 in 231 cells and Harrison et al, 2010 who established that Notch4 regulates TICs in breast cancers. Others have also demonstrated that Notch3 may play a role in breast cancer stem cell activity (Sansone et al., 2007).
3. Kim et al PNAS 2012; 109(16):6124-9 paper should be cited and the results discussed vis-à-vis the present findings.
4. The introduction cites Levina et al 2008 as a paper establishing that chemotherapy selectively enriches for TICs but this paper relates to lung cancer whereas the key paper in breast cancer is Li et al JNCI, 2008, and this should be cited instead.
5. Pannuti et al 2010 is cited as an example showing that Notch regulates breast cancer stem cells but citing original research papers rather than reviews would be more informative.

6. Semi-conservative replication is used several times but I am not sure this is the right term since it refers to chromosomal replication and segregation, which is not being proposed as the mechanism here. My suggestion is that asymmetric division is used in its place as it describes where one daughter cell retains a stem cell phenotype and one daughter cell differentiates.

Referee #3 (Remarks):

Summary:
The authors address an important question: are there subsets of tumor-initiating cells within tumors with distinct functional and molecular characteristics? Using established cell surface markers, the authors analyse subpopulations of cells from an established aggressive breast cancer cell line (MDA-MB-231) and two isolated primary human triple-negative breast carcinomas using functional in vitro and in vivo assays as well as biochemical analyses. The data presented to that extent yield insights into cellular heterogeneity within breast cancer cell lines and primary tumor material. This is an important topic in the field as cellular heterogeneity is directly related to therapeutic resistance.

However, while the authors identify subpopulations of breast cancer cells with different biological traits, the in vivo data do not support to label these subpopulations, as claimed in the title, breast cancer stem cells. The clarity of the manuscript could be greatly improved if some of the findings would be put more into a tumor heterogeneity/plasticity context. The in vivo data delivered by the authors strongly suggests differences between the isolated subsets with respect to proliferative capacity and metastasis, but do not convincingly reveal significant differences in tumor initiating ability.

Moreover, the authors suggest Notch as a therapeutic target in specific subpopulations of the aggressive MDA-MB-231 cell line and a primary tumor cell line. They are not the first to do so, indeed the Notch pathway was suggested to be a therapeutic target in MDA-MB-231 cells by Clementz AG. et al., Breast Cancer Res. 2011 Jun 14;13(3):R63. doi: 10.1186/bcr2900. However, what is novel and quite important is the author's stringent finding that the Notch pathway is specifically active in cell surface marker-defined subpopulations of tumor cells. Furthermore, the authors identify the transcription factor Sox 2 as a transcriptional target of Notch 1 and an effector of sphere-forming ability, a proxy assay of self renewal. However, for the latter results to be convincing, the authors need to differentiate between proliferation and self-renewal. More on this in the Figure-specific suggestions below.

The discussion of the manuscript reads strangely off-topic and should take into account the existing literature on NOTCH as a therapeutic target and, especially, on plasticity and the relevance of CD44 and CD24 as cancer stem cell markers for breast cancer. Works by others describing plasticity originating in CD24-negative cells (for ex. Chaffer C. et al., PNAS, 2011, Gupta PB. et al., Cell, 2011), as well as CD24-positive cells (Kim J. et al., PNAS 2012) should be discussed as well.

Figure 1.
- Figure 1D, 1G are redundant with 1E, 1H and can be omitted.
- With respect to Figure 1E-1I, it is not entirely clear how the assessment of ESA expression and ALDH1 activity fits in with the remainder of the manuscript, since this is something that is no further followed up. It is rather puzzling that the authors did not attempt to further delineate the CD24+ population within the two cell lines (MDA-MB-231 and DT-22), i.e., whether tumor-initiation cells could be further enriched by sorting cells that are double or even triple positive populations. As such, we only know that ESA+ and ALDH+ cells are present within the CD24+ population, but not the CD24- population. Therefore, the CD24+ population remains very heterogeneous. Further, in the absence of limited dilution assays to quantitate mammosphere formation (only once concentration of cells is used), there is no way of knowing which cells actually form the mammospheres. It's problematic to suggest further delineating these populations, since this would change the entire premise of the manuscript and require a re-doing of most of the experiments that follow. Within the scope of this manuscript, however, it would be very useful to determine,

whether tumor-initiating cells can be further enriched by comparing mammosphere formation of FACS sorted CD24+/ESA- vs. CD24+/ESA+ as well as CD24+/ALDH+/- or triple positive cells, at least from the MDA-MB-231 cell line. Further, to connect these data to the remainder of the manuscript, the authors should at least determine ESA-expression and ALDH1-activation in CD24+ cells treated with NOTCH-inhibitors, as done later.

Figure 4.
- From Figure 4A-C it is clear that tumor latency is longer and tumor growth is slower for CD24- compared to CD24+ cells sorted from MDA-MB-231 and DT-22 cells. However, from these data, it is not possible to discern differences in tumor initiation. Latency is not the same: for tumor initiation, the absence of formed tumors must be quantitated in a limited dilution assay. This necessitates the injection of cells into mice in decreasing number, but in Figure 4C, only one dilution is shown and and now quantification of the number of tumor-initiating cells in those populations cannot be provided based on the number of mice used. There might or might not be a difference. However, the fact that for both CD24- and CD24+ cells, 100 cells are sufficient to induce tumors in about half of the mice suggests that both populations are actually quite tumorigenic. That does not seem to be a decisive differentiating characteristic of these two populations: growth and metastasis however differ quite significantly, as shown in this figure
- Figure 4D. MDA-MB-231 cells metastasize spontaneously to many organs, including the lung. Therefore, it is puzzling that here, no lung metastasis can be detected. It is also known that MDA-MB-231 cells contain subpopulations with different metastasis patterns. Still, given that, separated into CD24- and CD24+ cells, all subpopulations contained within MDA-MB-231 cells where divided into two populations and then injected, one could have expected to observe at least one of these populations to effectively colonize the lungs, as MDA-MB-231 cells have been widely reported, and as has been the experience of the reviewer.

Another interesting issue the authors leave un-explored is the mechanism underlying the differential metastatic abilities of the CD24- vs. CD24+ cells. To this instant, it would be helpful to perform in vitro Boyden Chamber migration assays to determine whether they display different migratory ability. If this is the case, it would be further useful to test whether inhibition of NOTCH signaling has an impact on migration as well as self-renewal, i.e. whether these properties are linked via a common mechanism or not.

Figure 6./7.
- The inhibitory effects of NOTCH-inhibition and SOX2-knockdown on mammosphere formation would be much more convincing if, at the same time, the authors would provide growth curves in 2D cultures to be able to differentiate between general anti-proliferative/cytotoxic effects and specific effects on mammosphere formation. Especially in light of the fact that in vivo treatment also clearly shows antiproliferative effects. In vitro proliferation assays should therefore be included in the supplementary figures.

1st Revision - authors' response 07 June 2013

Reviewer # 1
General Remarks:

"the authors should have established a better correlation between the TNBC samples…. and the original tumors, ... how well did the original tumor reflect the phenotypes described in terms of marker and notch activation staining (frequency, would there be a way to identify patients subsets that might be eligible for clinical trials)?"

<u>Response:</u> Unfortunately, further comparison of primary tumor material with DT cultures are not immediately feasible since the IRB protocol at U Michigan over 5 yrs ago allowed tumor recovery for DT cultures but did not allow for subsequent retrieval and analysis of paraffin embedded primary tumor blocks. Thus further comparison of IHC for Notch and other markers in primary cancers and with corresponding levels in DTs is not possible. While data may ultimately support development of dual assays of N1-ICD and surface marker staining of dissociated primary tumors, the use of such staining as a predictive factor will require considerable work beyond the scope of this paper. The

results section now indicates that PAM50 breast cancer profiles were very similar in primary tumors and derived cultures. New data is added showing very early and later passage DTs revealed stable surface marker profiles (see also point 1 below).

"why would they insist on making a point on the metastatic potential of the CD44+/CD24low cells when this property seems to be unique to MDA-MB-231"

Response: Given this limitation of our data, as suggested, we decreased the emphasis on the metastatic potential of $CD44^{+}CD24^{low+}$ cells in the results and discussion. We made the striking observation that tumors formed from as few as 100 generated MDA-MB-231 $CD44^{+}CD24^{low+}$ cells metastasis cells, while up to 500,000 $CD44^{+}CD24^{neg}$ cells injected orthotopically failed to yield metastasis. Both DT-22 and MDA-MB-231 $CD44^{+}CD24^{low+}$ cells preferentially expressed gene profiles characteristic of breast cancers that metastasize to lung, brain and bone. Unfortunately, although the DT-22 model confirmed the $CD44^{+}CD24^{low+}$ population has a higher proportion of tumor initiating cells than $CD44^{+}CD24^{neg}$ (Fig. 3B), in further in vivo work that took over a year, we found DT-22 culture robustly form tumors that do not metastasize in either nude mice, Nod SCID or Nod SCID IL2-/- mice, with over 3 million cells injected. Repeating assays with other DTs would take over a year, extending well beyond this review. We added new data showing greater motility and invasion in $CD44^{+}CD24^{low+}$ cells of both DT-22 and MDA-MB-231, and toned down the emphasis on metastatic T-ISC heterogeneity.

The reviewer felt the connection between Notch 1 and Sox2 needed stronger supporting evidence.

Response: We believe data presented, as noted by other reviewers, shows that *SOX2*, a key mediator of ES self-renewal, is a novel transcriptional target of Notch 1 in these breast cancer models. An extended analysis of *NOTCH* genes in the revised paper showed *NOTCH* 1, 2 and 4 genes and their respective ICDs are more highly expressed in $CD44^{+}CD24^{low+}$, but only N1-ICD transfection upregulates Sox2. We show N1-ICD upregulated *SOX2* gene and protein expression and binds directly to the *SOX2* promoter using ChIP assays in two model systems. *SOX2* knockdown abrogated the effect of N1-ICD to enhance sphere formation (an in vitro proxy of self-renewal). New cell cycle and growth curve data confirm that increased sphere formation by N1-ICD transfection and loss thereof following *SOX2* knockdown do not merely reflect global effects on proliferation and support the notion that Notch1 critically up regulates *SOX2*, to promote $CD44^{+}CD24^{low+}$ cell self-renewal (Figure 4).

"why not focus on the characterization of the Roche Notch inhibitor, rather than DAPT, making it a more coherent study (move the Roche data to Figure 6/7 from supplement)"

Response: Per the reviewer's excellent suggestion, we focused on the characterization of the Roche Notch inhibitor RO4929097, and moved this data from supplementary to the main figures (See Figure 6A-D), and relocated the confirmatory data with the second GSI, DAPT to Supplemental data Fig. 6.

Specific points:

1) *"More details on cell passages, media utilized should be provided; in our own experience cell passaging affects dramatically TIC phenotypes"*

Response: As requested, we provide extended information on cell passages used and media utilized in the Materials and Methods. In over 25 DTs assayed, growth kinetics and CD44/CD24 surface staining have been very stable over extended passages as shown by new stable surface marker data for two DT cultures, DT-22 and DT-25 at passages 3-4 and 9-11 in revised Fig 1B and Supplementary Fig 2A. Furthermore, in the experiment where cells were repeatedly recovered and our two phenotypes flow sorted over 2-3 weeks in Fig 2B-E and Supplementary Fig 3, we tested the unsorted control (not shown). The marker distribution in unsorted cells recovered every 2 days over a period of 3 wks showed consistent marker expression in all of three repeated experiments for each of DT-22 and DT-25 and MDA-MB-231.

2) *"I would also ask the authors to refer to these two manuscripts:*

Specific inhibition of Notch1 signaling enhances the antitumor efficacy of chemotherapy in triple negative breast cancer through reduction of cancer stem cells.Qiu M, Peng Q, Jiang I, Carroll C, Han G, Rymer I, Lippincott J, Zachwieja J, Gajiwala K, Kraynov E, Thibault S, Stone D, Gao Y,

Sofia S, Gallo J, Li G, Yang J, Li K, Wei P. Cancer Lett. 2013 Jan 28;328(2):261-70. doi: 10.1016/j.canlet.2012.09.023. Epub 2012 Oct 3"
Characteristic genes in luminal subtype breast tumors with CD44+CD24-/low gene expression signature. Tsunoda Y, Sakamoto M, Sawada T, Sasaki A, Yamamoto G, Tachikawa T. Oncology. 2011;81(5-6):336-44. doi: 10.1159/000334690. Epub 2012 Jan 11"
Response: We now cited both references in the revised discussion.

Reviewer # 2
"Comments on Novelty/Model System: The strengths include use of patient-derived dissociated breast tumour cells to complement the cell line work, in vivo limiting dilution transplantation, and mechanistic insight into the regulation of the stem cell-like CD24+ population"
Specific points:
1) "The current paper reports that Notch inhibition prevents CD24+ to CD24- conversion but the outstanding question is can... Notch ligands induce CD24- to CD24+ interconversion."

Response: $CD24^{low+}$ cells showed Notch activation and GSI impaired sphere formation and tumor growth of $CD24^{low}$ but not $CD24^{neg}$ cells. While it is not feasible to stimulate the Notch pathway in these cultures by addition of Notch ligand, we tested if Notch1 activation by N1-ICD overexpression in these populations alter the phenotypes of progeny generated. We sorted N1-ICD overexpressing $CD24^{neg}$ and $CD24^{low+}$ populations and assayed CD24 expression in their respective progeny compared to vector controls over 15 days. New data (Fig 8) shows vector control $CD44^{+}CD24^{neg}$ cells yield only like progeny over 15 days as repeatedly observed in the course of this work. Notably, over-expression of N1-ICD in $CD44^{+}CD24^{neg}$ cells altered their phenotype, increasing the proportion of $CD24^{low+}$ cells generated from 0 to 13% (Fig. 8A & B) The proportions of $CD24^{low+}$ cells in the progeny of $CD44^{+}CD24^{low+}$ overexpressing N1-ICD was also significantly higher than their respective vector controls (Fig. 8C). Increased expression of N1-ICD levels was observed by Western blotting in both N1-ICD infected $CD24^{neg}$ and $CD24^{low+}$ sorted populations (Fig, 8D). Thus, Notch activation appears not only to increase the self-renewal/symmetric division of $CD24^{low+}$ cells but also to convert a small proportion of $CD24^{neg}$ to $CD24^{low+}$ within the 5-6 population doublings. Thus, this data indicate a potential conversion of CD24- to CD24+ when Notch1 is activated.

2) "Several times it is indicated that other Notch receptors are not involved for which we are shown little evidence and is conflicting with the literature,"

Response: We now include new data (Fig. 4B) showing relative mRNA expression of *NOTCH 1 to 4* in the unsorted and sorted populations of MDA-MB-231. Interestingly, *NOTCH 1, NOTCH 2,* and *NOTCH 4* expression and their respective ICDs were all increased in the $CD44^{+}CD24^{low+}$ than $CD44^{+}CD24^{neg}$. Notch1 was investigated further, since N1-ICD alone caused upregulation of Sox2 when overexpressed.

3) "Kim et al PNAS 2012; 109(16):6124-9 paper should be cited and the results discussed vis- à-vis the present findings"

Response: We have now cited this paper and modified our discussion to incorporate their important and relevant findings.

4) "The introduction cites Levina et al 2008 as a paper establishing that chemotherapy selectively enriches for TICs but this paper relates to lung cancer whereas the key paper in breast cancer is Li et al JNCI, 2008, and this should be cited instead."

Response: We have cited Li et al. JNCI, 2008 instead of Levina et al 2008, as requested.

5) "Pannuti et al 2010 is cited as an example showing that Notch regulates breast cancer stem cells but citing original research papers rather than reviews would be more informative"

Response: We have now expanded the review of Notch in breast cancer and breast cancer stem cells in the discussion and cited original papers.

6) "Semi-conservative replication is used several times but I am not sure this is the right term since it refers to chromosomal replication and segregation, which is not being proposed as the mechanism here. My suggestion is that asymmetric division is used in its place as it describes where one daughter cell retains a stem cell phenotype and one daughter cell differentiates"

Response: We thank the reviewer and have replaced "semi-conservative" with "asymmetric division".

Reviewer # 3
General Remarks:

"The authors address an important question: are there subsets of tumor-initiating cells within tumors with distinct functional and molecular characteristics? .... This is an important topic in the field as cellular heterogeneity is directly related to therapeutic resistance.
However, while the authors identify subpopulations of breast cancer cells with different biological traits, the in vivo data do not support to label these subpopulations…breast cancer stem cells. .. clarity ... could be greatly improved if some of the findings would be put more into a tumor heterogeneity/plasticity context….The in vivo data delivered by the authors strongly suggests differences between the isolated subsets with respect to proliferative capacity and metastasis, but do not convincingly reveal significant differences in tumor initiating ability"
Response: Additional limiting dilution tumor formation data with the DT-22 model presented in Fig 3A, B (revised Fig 4—see point 2 below) show statistically significant differences in the frequency of tumor-initiating cells between the $CD24^{neg}$ and $CD24^{low}$. Furthermore, new limiting dilution sphere analysis of $CD44^{+}CD24^{neg}$ and subsets of $CD44^{+}CD24^{low+}$ cells in revised Figure 7 (see point 1 below) also provide in vitro proxy assays that indicate self-renewal differences in these marker-defined subpopulations in the MDA-MB-231 model. Moreover, further analysis of growth curves and cell cycle profiles of these subsets with and without GSI, N1-ICD overexpression and Sox2 knockdown indicate that findings are more likely attributable to differences in a stem-like populations than mere differences in proliferation (see point 4 below). Nonetheless we have, as requested, attempted to situate our data within a context of tumor heterogeneity/plasticity in the significantly revised discussion but also retain the notion that present data support both heterogeneity and precursor progeny relationships between tumor-initiating subsets within triple-negative breast cancers. We changed the title to remove stem cells.

"The authors identify the transcription factor Sox 2 as a transcriptional target of Notch 1 and an effector of sphere-forming ability, a proxy assay of self renewal. However, for the latter results to be convincing, the authors need to differentiate between proliferation and self-renewal. More on this in the Figure-specific suggestions below"

Response: We thank the reviewer for highlighting critical data needed to rule out simple differences in proliferation between populations. For response please see Point 6 below.

"The discussion of the manuscript reads strangely off-topic and should take into account the existing literature on NOTCH as a therapeutic target and, especially, on plasticity and the relevance of CD44 and CD24 as cancer stem cell markers for breast cancer. Works by others describing plasticity originating in CD24-negative cells (for ex. Chaffer C. et al., PNAS, 2011, Gupta PB. et al., Cell, 2011), as well as CD24-positive cells (Kim J. et al., PNAS 2012) should be discussed as well"

Response: We have modified the discussion to include greater focus on NOTCH as a therapeutic target and regulator of breast cancer stem-like cells and on the emerging data from other groups supporting the existence of both heterogeneity (with or without hierarchy) and plasticity within tumor initiating cells.

Specific points:
1) "Figure 1D, 1G are redundant with 1E, 1H and can be omitted."

Response: We have moved data in prior Fig1 D and G to supplemental data.

2) "it would be very useful to determine, whether tumor-initiating cells can be further enriched by comparing mammosphere formation of FACS sorted CD24+/ESA- vs. CD24+/ESA+ as well as CD24+/ALDH+/- or triple positive cells, at least from the MDA-MB-231 cell line. Further,

to connect these data to the remainder of the manuscript, the authors should at least determine ESA-expression and ALDH1-activation in CD24+ cells treated with NOTCH-inhibitors, as done later"

Response: The analysis of these subpopulations within $CD44^{+}CD24^{low+}$ group revealed additional levels of heterogeneity within TNBC stem-like cells. As requested, we have sorted the $CD24^{low+}$/ESA+ vs $CD24^{low+}$/ESA- as well as $CD24^{low+}$ALDH1+ vs $CD24^{low+}$ALDH1- from the MDA-MB-231 line and performed limiting dilution mammosphere assays (See revised Fig. 7A & B). The proportion of sphere forming cells in the $CD24^{low+}$ESA+ subset was modestly but significantly higher than in $CD24^{low+}$ESA-. Sphere initiating cell abundance was also higher in the $CD24^{low+}$ALDH1+ cells than in the $CD24^{low+}$ALDH1- subpopulation. GSI treatment decreased the proportion of sphere forming cells in all $CD24^{low+}$subpopulations but not in $CD24^{neg}$ cells. Notably, $CD24^{low+}$ALDH1+ cells appeared to be most sensitive to GSI, suggesting heterogeneity in GSI responsiveness even within the $CD24^{low+}$ subset. Triple sorting of putative $CD24^{low+}$ESA+,ALDH1+ populations was not technically feasible hence the relationship between ESA+ and ALDH1+ subgroups remains unclear.

3) "From Figure 4A-C it is clear that tumor latency is longer and tumor growth is slower for CD24- compared to CD24+ cells sorted from MDA-MB-231 and DT-22 cells… from these data, it is not possible to discern differences in tumor initiation. Latency is not the same: for tumor initiation, the absence of formed tumors must be quantitated in a limited dilution assay. This necessitates the injection of cells into mice in decreasing number, but in Figure 4C, only one dilution is shown ... quantification of the number of tumor-initiating cells in those populations cannot be provided based on the number of mice used. ...growth and metastasis however differ quite significantly, as shown in this figure"

Response: Additional limiting dilution tumor formation data with the DT-22 model (new Fig 3A, B, formerly Fig 4) now show not only differences in tumor latency, but also show statistically significant differences in the frequency of tumor-initiating cells between the $CD24^{neg}$ and $CD24^{low}$. The tumor-initiating frequency (Figure 3B) calculated with L-Calc software show tumor-initiating cells in 1/44,936 $CD24^{neg}$ compared to 1 in 72 $CD24^{low+}$. With MDA-MB-231 at 100 cells injected, both populations were quite tumorigenic. In vivo quantitation of frequency of tumor-initiating cells from 10 MDA-MB-231 cell injections would take 9-12 months and we hope the reviewers will agree that this significant delay in publication is not warranted given the strength and importance of the work as a whole, the significant difference observed between the two populations sorted from the primary dissociated DT-22 model and new limiting dilution sphere analysis of $CD44^{+}CD24^{neg}$ and subsets of $CD44^{+}CD24^{low+}$ cells in revised Figure 7 (see point 1 below) that provide in vitro proxy assays that indicate self-renewal differences in these MDA-MB-231 subpopulations.

4) "Figure 4D. MDA-MB-231 cells metastasize spontaneously to many organs, including the lung.., it is puzzling that here, no lung metastasis can be detected. It is also known that MDA-MB-231 cells contain subpopulations with different metastasis patterns. Still, given that, separated into CD24- and CD24+ cells, all subpopulations contained within MDA-MB-231 cells where divided into two populations and then injected, one could have expected to observe at least one of these populations to effectively colonize the lungs, as MDA-MB-231 cells have been widely reported, and as has been the experience of the reviewer"

Response: The luciferase tagged line used in this work was obtained from the Massague lab and has been compared with their more highly metastatic variants. It is worth pointing out that while overt lung metatstasis were not detected by IVIS, micrometastasis in lung tissue were readily detected after orthotopic injection of $CD44^{+}CD24^{low+}$ but not in animals injected with $CD44^{+}CD24^{low+}$(see discussion).

5) *"Another interesting issue the authors leave un-explored is the mechanism underlying the differential metastatic abilities of the CD24- vs. CD24+ cells. To this instant, it would be helpful to perform in vitro Boyden Chamber migration assays to determine whether they display different migratory ability…….(and) to test whether inhibition of NOTCH signaling has an impact on migration as well as self-renewal"*

Response: As requested, we tested real-time migration and invasion potential of the CD24- vs CD24+ cells by Xcelligence real time analysis with and without GSI (Fig. 6E and Supplementary Fig S6G). CD24+ cells showed greater transwell migration and greater invasive ability than the

CD24- cells. Notch inhibition significantly attenuated the ability of CD24+ cells to migrate and invade matrigel while the $CD24^{neg}$ were unaffected.

6) "The inhibitory effects of NOTCH-inhibition and SOX2-knockdown on mammosphere formation would be much more convincing if ... the authors would provide growth curves in 2D cultures to ... differentiate between general anti-proliferative/cytotoxic effects and specific effects on mammosphere formation."

Response: As suggested, we now show that cell cycle profiles were not changed in any cell group after 48 hours of GSI treatment (Fig. 6F and supplemental Fig S6H) and growth curves in 2D cultures were not affected by GSI over several days in any of the sorted populations (Supplementary Fig S6 I-J). Moreover, cell cycle profiles and growth curves were unchanged by either N1-ICD-transfection or *SOX2*-knockdown (Fig. 5I & J). Thus, the effects of Notch inhibitors, N1-ICD overexpression and *SOX2* knockdown on sphere formation were not merely attributable to effects on cell proliferation and support specific effects on sphere forming cells.

We look forward to your response and thank you for your time and consideration.

2nd Editorial Decision 08 July 2013

Thank you for the submission of your revised manuscript to EMBO Molecular Medicine. We have now received the enclosed reports from the referees that were asked to re-assess it. As you will see the reviewers are now globally supportive and I am pleased to inform you that we will be able to accept your manuscript pending the following final amendments:

1) The quality of the current figure images and text is too low (they both appear rather blocky/blurry). Please provide higher resolution versions, and check to make sure that text/line-art remains clear even when zooming in (which is not currently the case). You may find that saving the images as EPS or PDF will better preserve the text and line-art resolution. If this does not help, you may need to remake the figures in a quality vector graphics program like Illustrator or the free opensource, alternative Inkscape.

2) Reviewer 3 notes that the error bars are missing from all of the new in vitro growth curves and should thus be added.

Please submit your revised manuscript within two weeks. Needless to say, the sooner you send us the revised, final version, the sooner I will be able to accept it for publication.

***** Reviewer's comments *****

Referee #1 (Comments on Novelty/Model System):

remain somewhat concerned by the point made around increased metastatic potential of cd44+/cd24low cells, this being only proven in MDA231 cells.

Referee #2 (Comments on Novelty/Model System):

The technical quality has improved in the revised MS and it remains a novel finding that has potential clinical impact.

Referee #2 (Remarks):

The authors have responded adequately to my comments and other reviewers and the MS is now markedly improved from its first iteration and highly suitable for publication.

Referee #3 (Remarks):

The authors have responded to all of the points raised by the reviewers and addressed all the important ones very carefully. At the same time, they have taken care to strengthen the main messages of the manuscript, which has been substantially improved in clarity and impact.

As a minor point to be addressed: error bars are missing from all of the newly done in vitro growth curves and should be added.

2nd Revision - authors' response 15 July 2013

We are submitting a revised final version of our manuscript entitled **"Triple negative breast cancer initiating cell subsets differ in functional and molecular characteristics and in γ-secretase inhibitor drug responses".**
We now provide higher resolution PDF figures and show error bars in the new in vitro growth curves as suggested by reviewer 3.
