# Supplementary material for: Triple negative breast cancer initiating cell subsets differ in functional and molecular characteristics and in γ-secretase inhibitor drug responses
Source: EMBO Mol Med. 2013 Aug 27;5(10):1502–22. doi: 10.1002/emmm.201302558 (PMC3799576; doi:10.1002/emmm.201302558)
Supplement: Supplementary file 2 [file emmm0005-1502-SD2.pdf]

# Supplemental Information - Table of content:

**SUPPLEMENTAL FIGURES AND LEGENDS**....................................................................**2**

**FIGURE S1:** FREQUENT OBSERVATION OF CD44$^{+}$CD24$^{NEG/LOW}$ SURFACE EXPRESSION IN ER NEGATIVE BREAST CANCER LINES AND TUMOR CULTURES....................................................................2

**FIGURE S2:** ESA EXPRESSION AND ALDEFLUOR ACTIVITY ARE OBSERVED IN SUBSETS OF CD44$^{+}$CD24$^{LOW+}$ CELLS.................................................................... 3

**FIGURE S3:** CD44$^{+}$CD24$^{LOW+}$ GIVE RISE TO BOTH CD44$^{+}$CD24$^{LOW+}$ AND CD44$^{+}$CD24$^{NEG}$ PROGENY WHILE CD44$^{+}$CD24$^{NEG}$ YIELD ONLY CD44$^{+}$ CD24$^{NEG}$ .................................................................... 4

**FIGURE S4:** EXPRESSION OF LUNG, BRAIN AND BONE METASTASIS GENE EXPRESSION SIGNATURES IS ENRICHED IN CD44$^{+}$CD24$^{LOW+}$ SUBPOPULATIONS FROM MDA-MB-231 AND DT-22.................................................................... 5

**FIGURE S5:** EXPRESSION OF EMBRYONIC STEM CELL TRANSCRIPTION FACTORS AND TARGETS IS ENRICHED IN CD44$^{+}$CD24$^{LOW+}$ SUBPOPULATIONS.................................................................... 7

**FIGURE S6:** GSI SENSITIVITY IS RESTRICTED TO THE CD44$^{+}$CD24$^{LOW+}$ .................................................................... 9

**LEGENDS FOR TABLE S1 (DATASET)**....................................................................**11**

**TABLE S1:** INDIVIDUAL GENE SCORES FROM GSA IN SORTED CD44$^{+}$CD24$^{LOW+}$ COMPARED TO CD44$^{+}$CD24$^{NEG}$ SUBPOPULATIONS FROM MDA-MB-231 AND DT-22 CELLS....................................................................11

**SUPPLEMENTAL METHODS**....................................................................**12**

CHROMATIN IMMUNOPRECIPITATION ASSAY....................................................................12

REAL TIME PCR PRIMER SEQUENCES....................................................................12

MICROARRAY DATA ACQUISITION AND ANALYSIS....................................................................12

MICROARRAY DATA PROCESSING AND NORMALIZATION....................................................................12

DEFINING A NOTCH TARGET GENE SET....................................................................13

GENE SET ENRICHMENT ANALYSIS....................................................................13

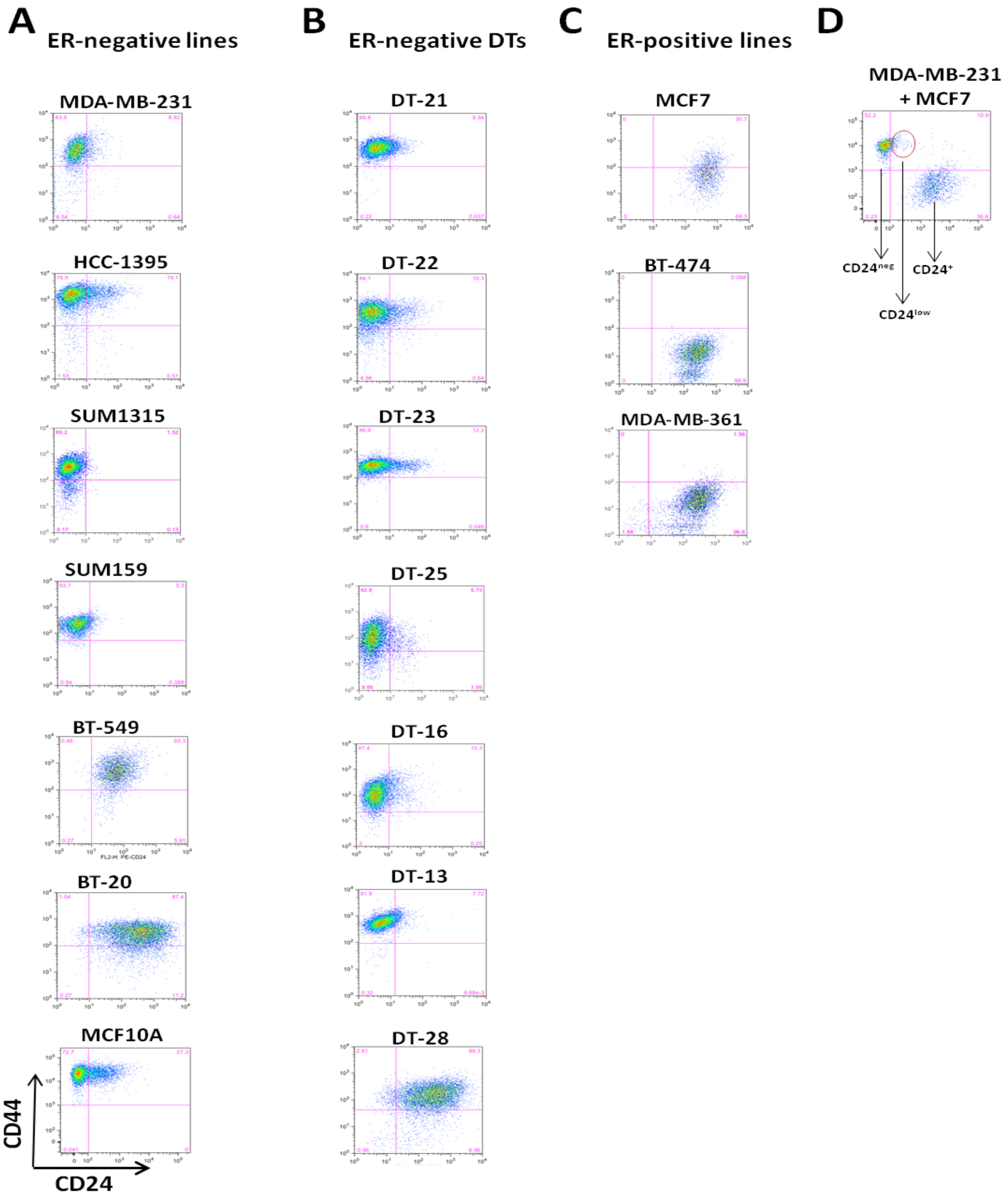


**Figure S1: Frequent observation of CD44$^{+}$CD24$^{neg/lo}$ surface expression in ER negative breast cancer lines and tumor cultures.**

**A.** ER negative breast cancer cell lines
**B.** TNBC-derived dissociated tumor (DT) cultures
**C.** ER positive lines and
**D.** Equal numbers of mixed MDA-MB-231 and MCF7 cells.

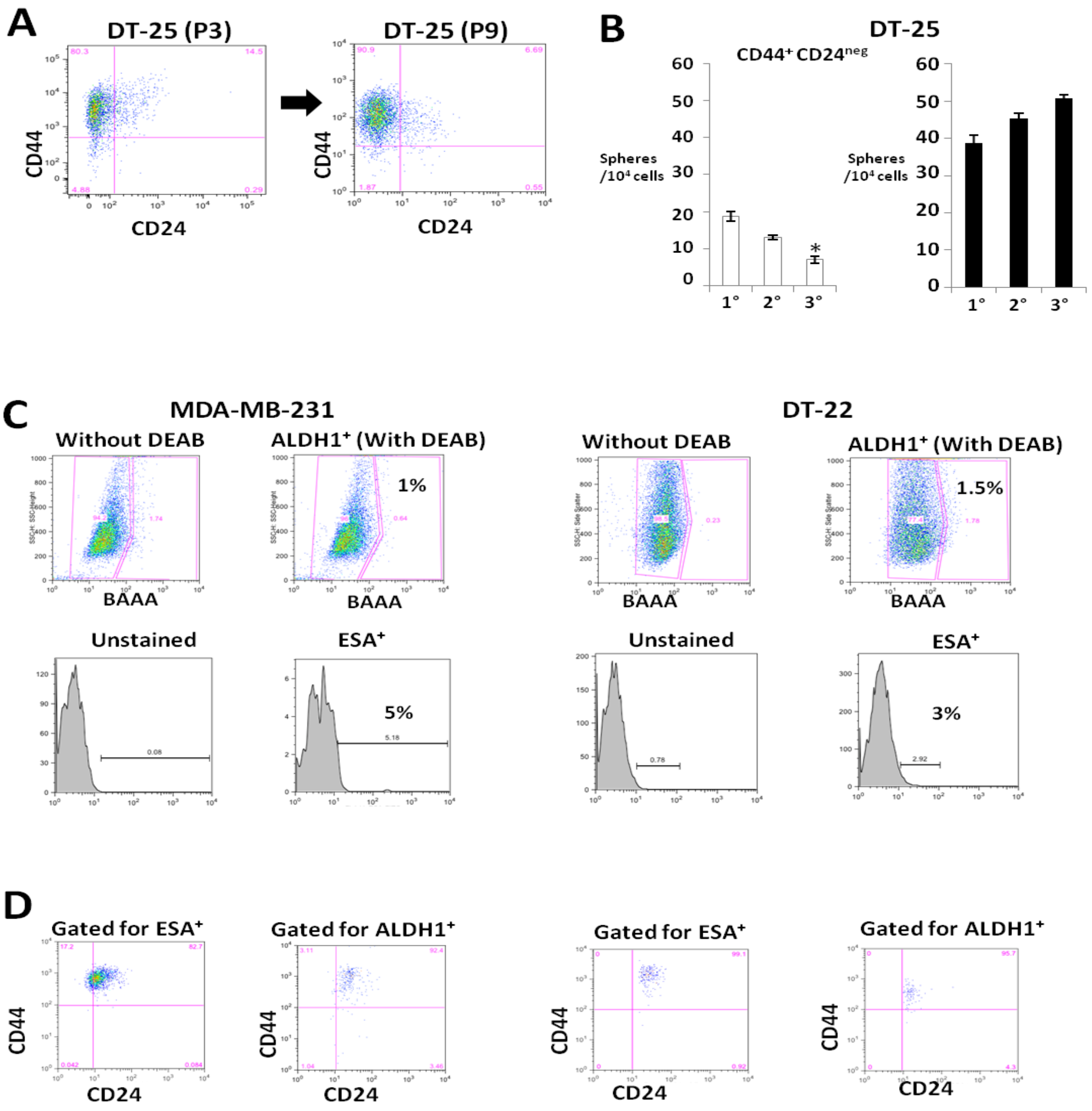


**Figure S2. ESA expression and aldefluor activity are observed in subsets of CD44$^+$CD24$^{low+}$ cells.**

**A.** CD44 and CD24 in DT-25 at passage (P3) compared to DT-25 at passage (P9).

**B.** Serial mammospheres formed from sorted CD44$^+$CD24$^{low+}$ and CD44$^+$CD24$^{neg}$ from DT-25**.** Mean +/- SEM, *p=0.0054

**C.** Aldefluor activity (top) and flow cytometry for surface ESA (bottom) were assayed as described in MDA-MB-231 and DT-22. Respective unstained controls are shown.

**D.** Cells were gated for ESA$^+$ (left) or ALDH1$^+$ (right) and CD44/CD24 assayed.

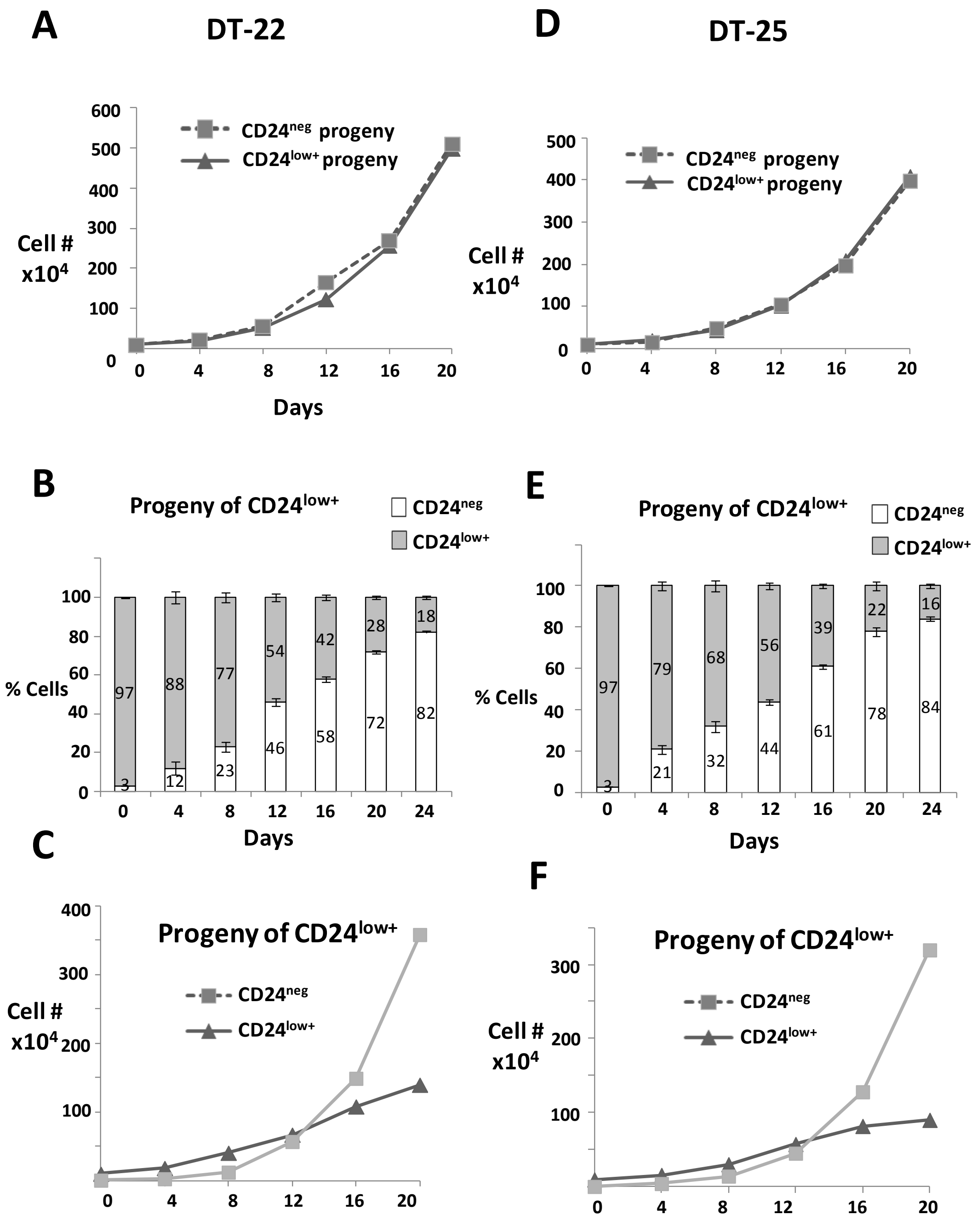


**Figure S3. CD44$^{+}$CD24$^{low+}$ give rise to both CD44$^{+}$CD24$^{low+}$ and CD44$^{+}$CD24$^{neg}$ progeny while CD44$^{+}$CD24$^{neg}$ yield only CD44$^{+}$CD24$^{neg}$.**

CD44$^{+}$CD24$^{low+}$ or CD44$^{+}$CD24$^{neg}$ were sorted from DT-22 and DT25 and 100,000 cells cultured.

**A, D.** Population growth from sorted CD44$^{+}$CD24$^{low+}$ and CD44$^{+}$CD24$^{neg}$

**B, E.** The proportion of CD44$^{+}$CD24$^{low+}$ or CD44$^{+}$CD24$^{neg}$ cells arising from CD44$^{+}$CD24$^{low+}$ cells is shown over time.

**C, F.** Growth curves of progeny of CD44$^{+}$CD24$^{low+}$ over 14 days. As for MDA-MB-231 (Figure 3A) CD44$^{+}$CD24$^{neg}$ generated only CD44$^{+}$CD24$^{neg}$ cells over 14 days (not shown here).

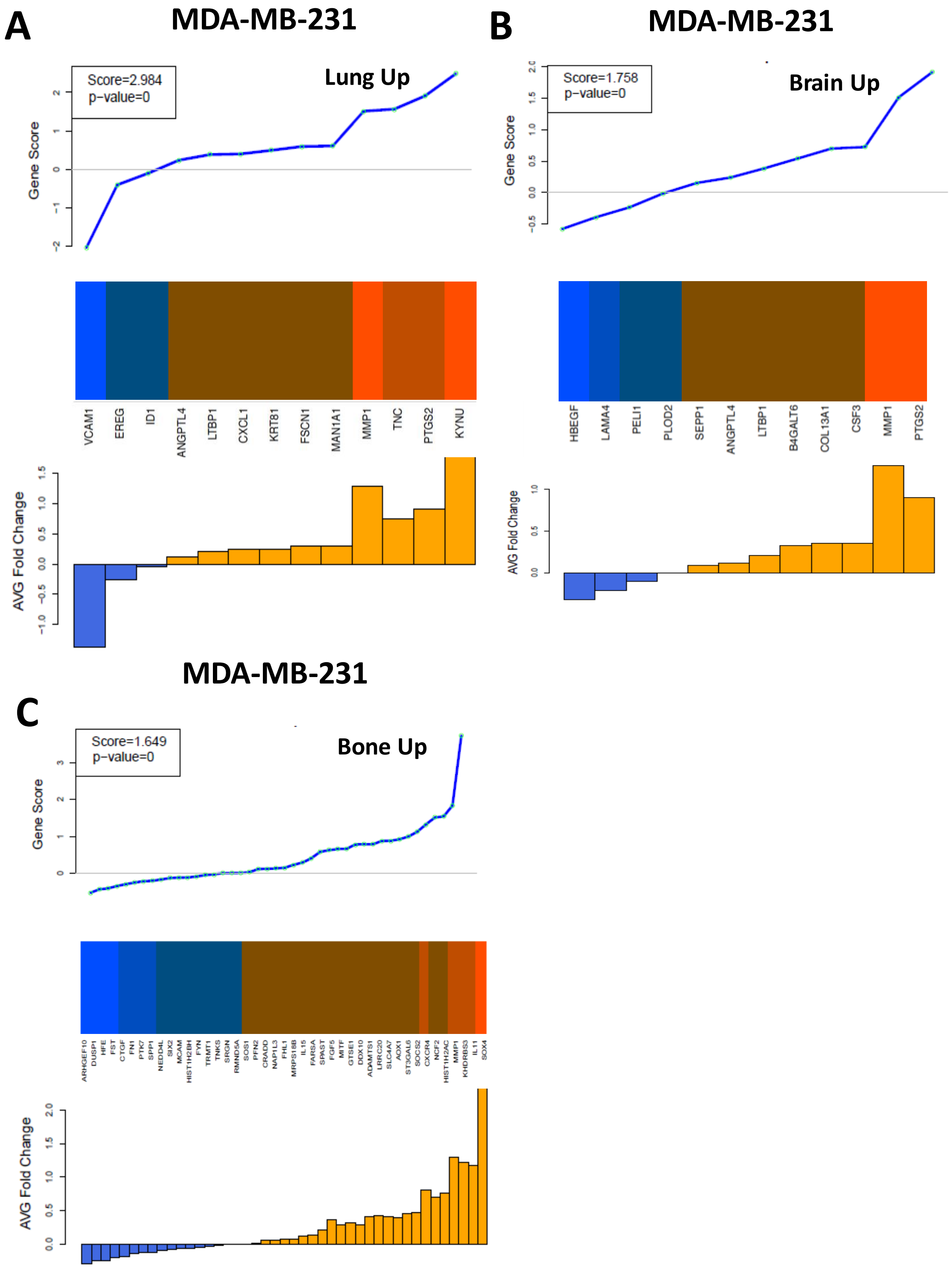

A
MDA-MB-231
Score=2.984
p-value=0
Lung Up
Gene Score
VCAM1
EREG
ID1
ANGPTL4
LTBP1
CXCL1
KRT81
FSCN1
MAN1A1
MMP1
TNC
PTGS2
KYNU
AVG Fold Change
B
MDA-MB-231
Score=1.758
p-value=0
Brain Up
Gene Score
HBEGF
LAMA4
PELI1
PLOD2
SEPP1
ANGPTL4
LTBP1
B4GALT6
COL13A1
CSF3
MMP1
PTGS2
AVG Fold Change
MDA-MB-231
C
Score=1.649
p-value=0
Bone Up
Gene Score
ARHGEF10
DUSP1
HFE
FST
CTGF
FN1
PTK7
SPP1
NEDD4L
SIX2
MCAM
HIST1H2BH
FYN
TRMT1
TNKS
SRGN
RMND5A
SOS1
PFN2
CRADD
NAP1L3
FHL1
MRPS18B
IL15
FARSA
SPAST
FGF5
MITF
GTSE1
DDX10
ADAMTS1
LRRC20
SLC4A7
AOX1
ST3GAL6
SOCS2
CXCR4
NCF2
HIST1H2AC
MMP1
KHDRBS3
IL11
SOX4
AVG Fold Change

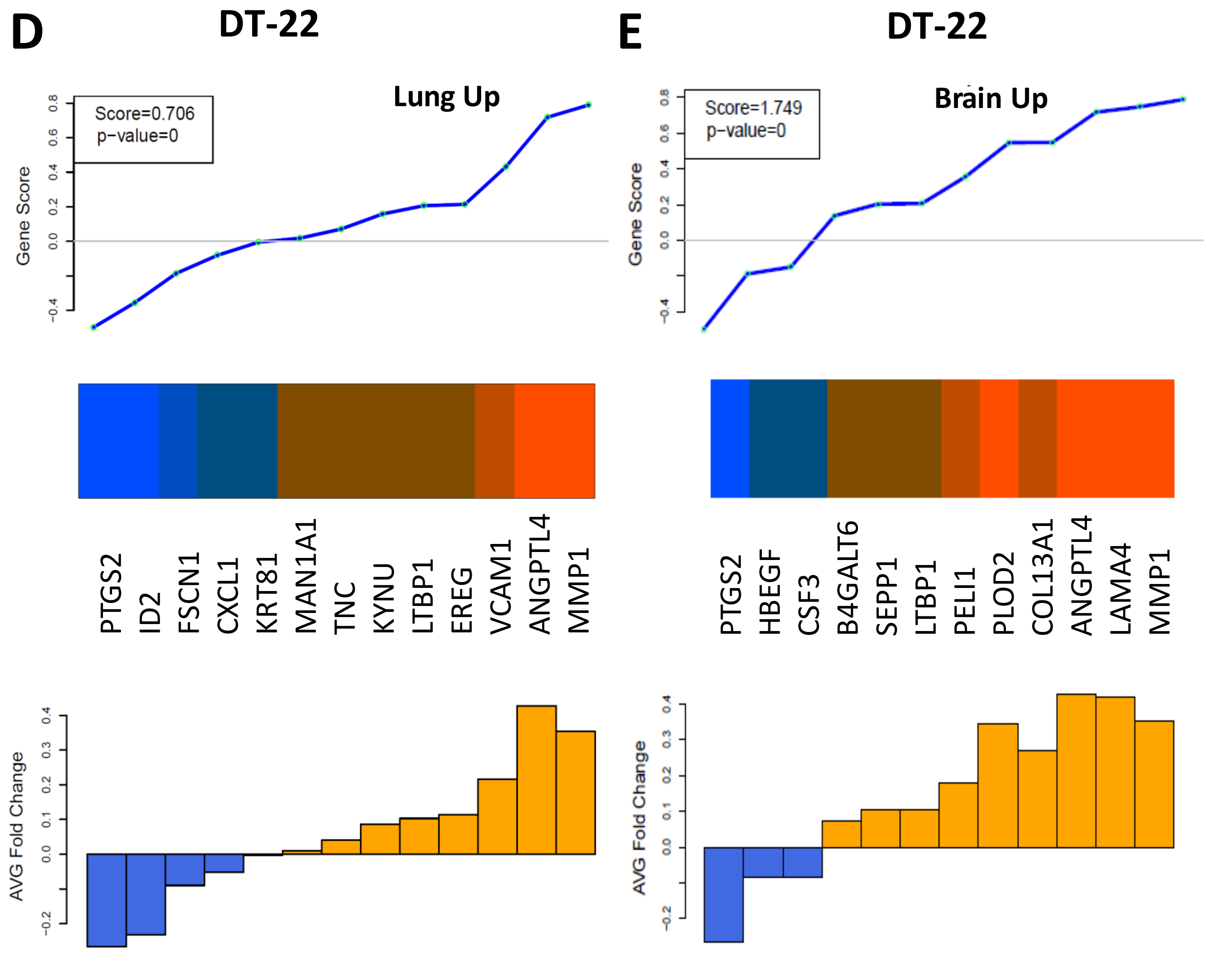


**Figure S4. Expression of lung, brain and bone metastasis gene expression signatures is enriched in $CD44^{+}CD24^{low+}$ subpopulations from MDA-MB-231 and DT-22**

**A,B,C.** Expression of lung **(A)**, brain **(B)** and bone **(C)** metastasis gene expression signatures is enriched in $CD44^{+}CD24^{low+}$ subpopulations in MDA-MB-231. Gene set analysis (GSA) compared enrichment of indicated signatures. Shown are ordered gene scores for each gene in the line plot and the average fold change in the heatmap (orange indicates high expression in $CD44^{+}CD24^{low+}$ and blue is low). Average fold gene expression changes are indicated by bar graphs. Upper left panel shows enrichment score and the p-value.

**D,E.** Expression of lung **(D)** and brain **(E)** metastasis gene expression signatures is enriched in $CD44^{+}CD24^{low+}$ subpopulations in DT-22 analysis as above. A list of the genes used for each signature and their individual gene scores are in shown in Supporting Information Table 1.

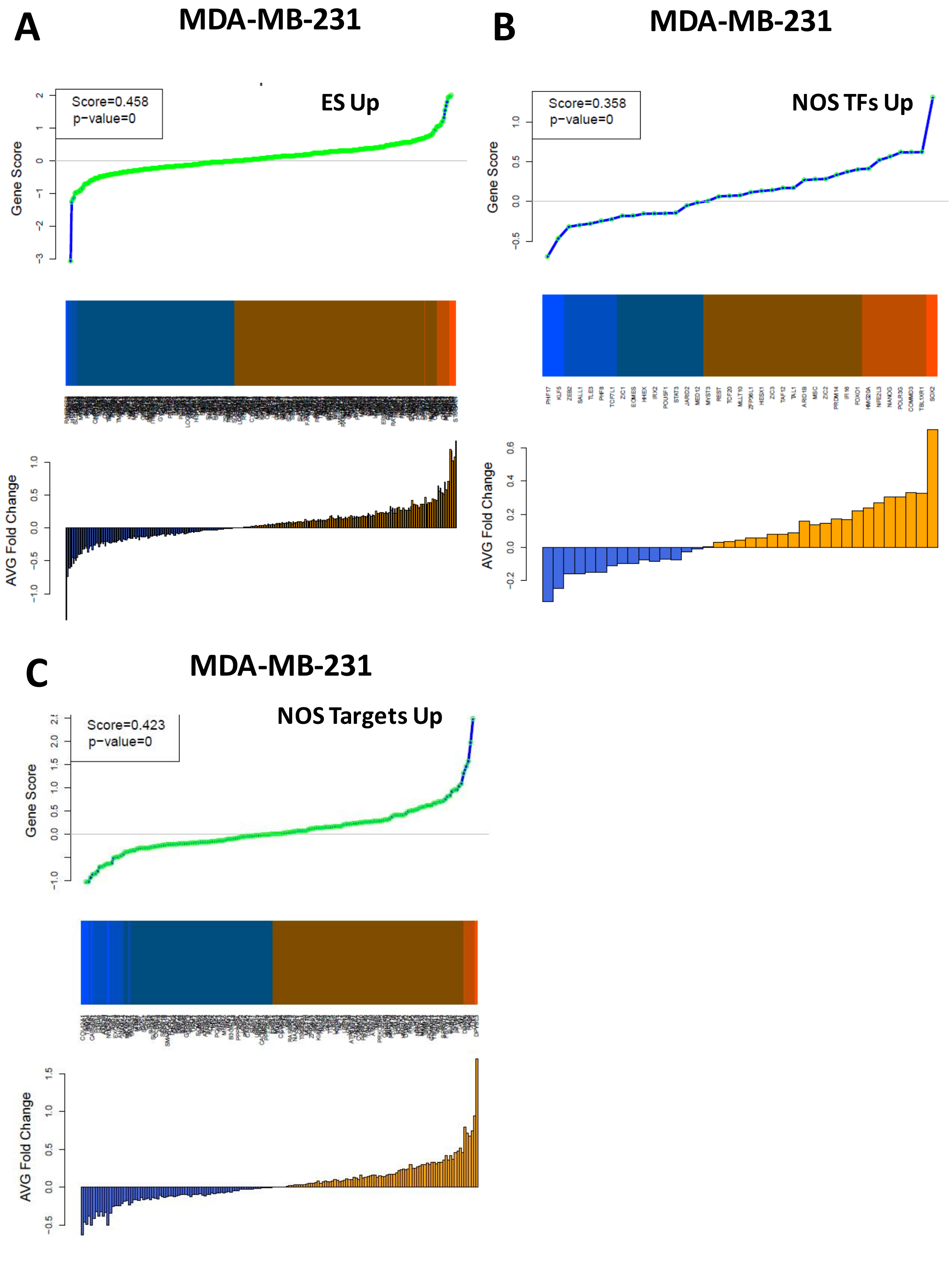
A
MDA-MB-231
Score=0.458
p−value=0
ES Up
Gene Score
AVG Fold Change
B
MDA-MB-231
Score=0.358
p−value=0
NOS TFs Up
Gene Score
AVG Fold Change
C
MDA-MB-231
Score=0.423
p−value=0
NOS Targets Up
Gene Score
AVG Fold Change

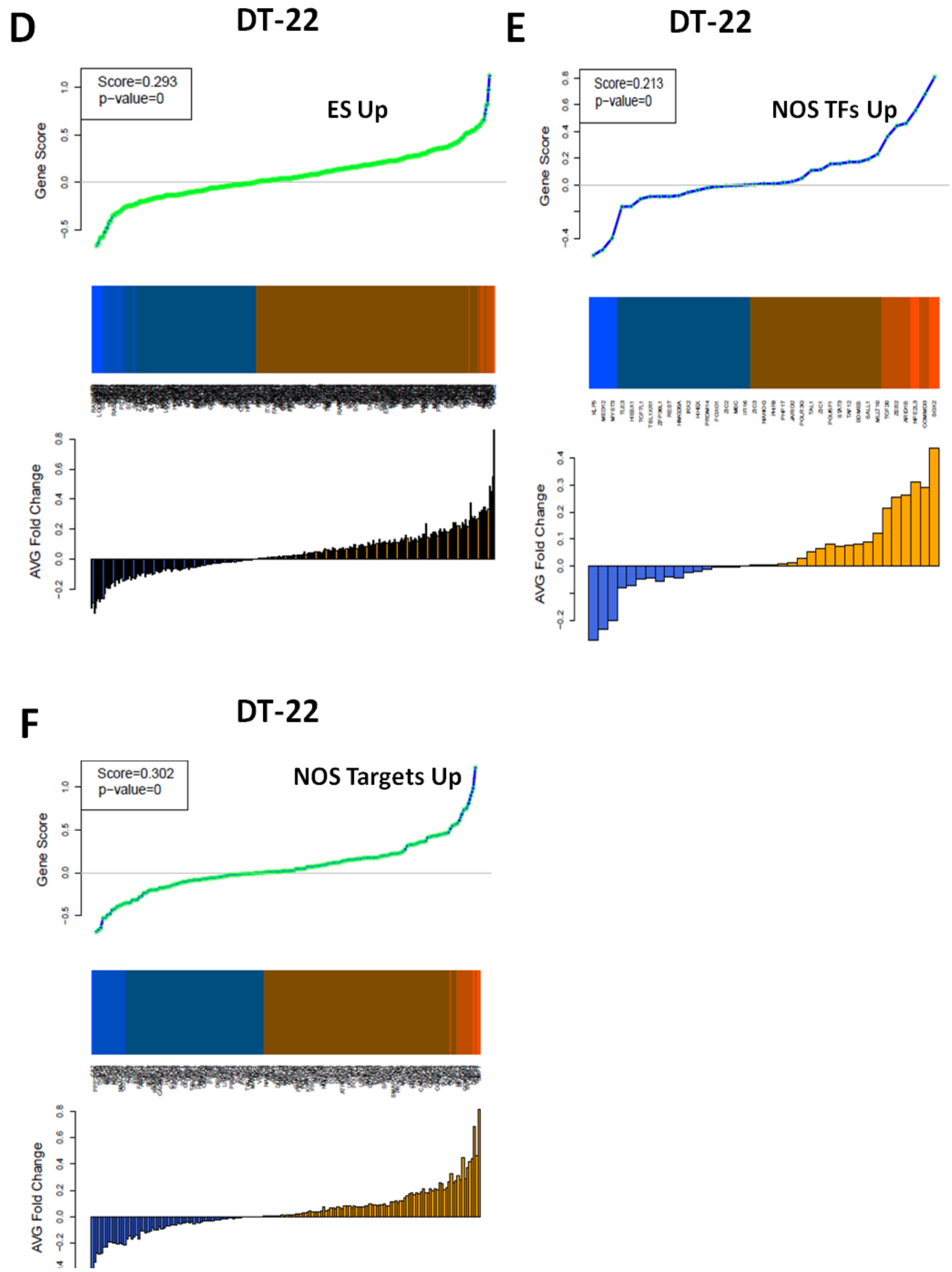


**Figure S5. Expression of embryonic stem cell transcription factors and targets is enriched in CD44$^+$CD24$^{low+}$ subpopulations.**

**A-F.** GSA comparing enrichment of indicated embryonic stem cell transcription factors **(A, B** and **D, E)** and genes upregulated by NOS (Nanog, Oct4 and Sox2) **(C, F)**. The “NOS TF up” is a gene set of transcriptional regulators identified in human ES that overexpress Nanog, Oct4, or Sox2. The “NOS Targets up” gene set contains the activated genes from the ChIP-array for Nanog, Oct4, and Sox2. A list of the genes used for each signature and their individual gene scores are in shown in Supplementary Table 1. For MDA-MB-231 **(A-C)**, and DT-22 **(D-F)** the ordered scores for each gene in the line plot and the average fold change in the heatmap are shown (orange indicates high expression in CD44$^+$CD24$^{low+}$ and blue, low). Enrichment scores and p-values are at upper left. Average fold gene expression changes are shown in bar graphs and a list of genes and their individual gene scores are in Supporting Information Table 1.

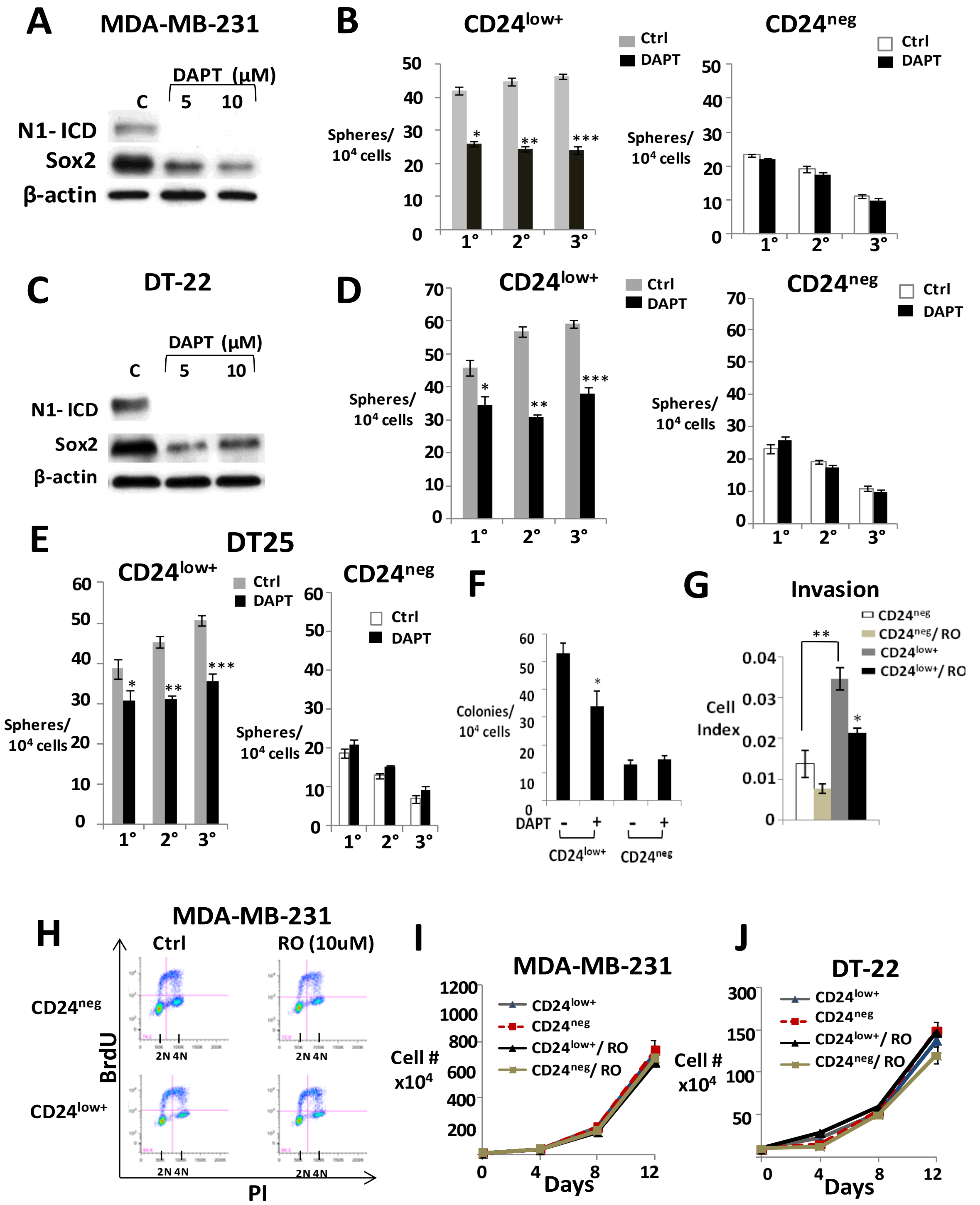


**Figure S6. GSI sensitivity is restricted to the CD44$^{+}$CD24$^{low+}$.**

**A- E.** CD44$^{+}$CD24$^{low+}$ (CD24$^{low+}$) and CD44$^{+}$CD24$^{neg}$ (CD24$^{neg}$) cells sorted from MDA-MB-231 **(A, B)**, DT-22 **(C, D)** and DT-25 **(E)** were treated with or without DAPT. Effect of DAPT on cleaved Notch 1 (N1-ICD) and Sox2 +/- after 24 hrs of 5 and 10µM DAPT in CD44$^{+}$CD24$^{low+}$ cells **(A & C)**. Serial mammospheres of indicated cells from sorted populations +/- 5µM DAPT (Mean ± SEM, Student's *t*-test). The p values in panel B: *p= 0.00001; **p=0.00002; ***p=0.0001; panel D: *p= 0.0045; **p=0.006; ***p=0.0054; panel

E: *p= 0.0027; **p=0.0005; ***p=0.007 Serial mammospheres from DAPT-treated $CD24^{low+}$ cells were significantly reduced compared to respective untreated controls. (*) denotes means statistically different by student's *t* test from control primary spheres. Treated and untreated tertiary mammospheres from $CD24^{neg}$ cells were reduced significantly compared to primary spheres, but both were unaffected by DAPT **(B, D & E)**.

**F.** Mean soft agar colonies arising from sorted populations of MDA-MB-231 +/- 5μM DAPT (Mean +/- SEM, t test, *p=0.04).

**G.** Matrigel invasion of sorted populations from DT-22 +/- 10μM RO4929097 (RO) generated by xCELLigence real time cell analysis, graphed as Mean ± SEM. $CD24^{low+}$ cells show significantly greater invasion compared to $CD24^{neg}$ cells (**p= 0.0055 at T=12 hrs, Student's *t* test). RO significantly attenuated invasion (*p= 0.004 at T= 12hrs, Student's *t* test) of $CD24^{low+}$ cells. $CD24^{neg}$ cells were unaffected.

**H.** Cell cycle profiles of sorted populations from MDA-MB-231 after 48 hrs +/- 10μM RO4929097.

**I, J.** GSI does not affect MDA-MB-231 or DT-22 cell proliferation. Proliferation curves of 100,000 cells from sorted $CD44^{+}CD24^{low+}$ or $CD44^{+}CD24^{neg}$ populations from MDA-MB-231 **(I)** and DT22 **(J)** cultured over 12 days +/- 10μM RO4929097.

## Legend for Table S1 (Dataset)

**Table S1:** Individual gene scores from GSA results showing enrichment of various gene signatures in sorted $CD44^{+}CD24^{low+}$ compared to $CD44^{+}CD24^{neg}$ subpopulations from MDA-MB-231 and DT-22 cells. Higher gene scores indicate increased expression while lower gene scores indicate decreased expression.

## Supplemental Methods

**Chromatin Immunoprecipitation Assay:**
Cells were treated with 2% formaldehyde for 10 min at $22^0$C for ChIP assay with anti-cleaved Notch1 antibody (Cell Signaling) or control IgG. Immunoprecipitated DNA corresponding to –770 to –616 from the transcriptional start site of human Sox 2 promoter was amplified by PCR using the primer pair:
SOX2-F: GCCAAAGAGCTGAGTTGGAC; SOX2-R :CCCAAACCTCTGTCCTCAAA
The two regions of the mouse Sox2 promoter were amplified by PCR using the following primer pairs:
mSOX2(1)-F:CTGTGGTTGCTCTTTGTAGCA;
mSOX2(1)-R:TGTAGGGGCACCTTCATTTT;
mSOX2(2)-F:CCTAGGAAAAGGCTGGGAAZ;
mSOX(2)-R: CACTCACCCCCTCTTCTCAC.

**Real Time PCR Primer Sequences:**
The primers used to amplify NOTCH1, NOTCH2, NOTCH3, NOTCH4, NANOG, SOX2, JAGGED1, HEY1, GAPDH and HPRT (F = forward; R= reverse) were as follows:
hNotch1-F: GAAGAACGGGGCTAACAAAGAT;
hNotch1-R: GTCCATATGATCCGTGATGTCC;
hNotch2-F: AGCTACTGTGAGGAGCAACTCG;
hNotch2-R: GATTCTGGCACTCATCCACTTC;
hNotch3-F: TCAAAAATGGAGCCAATAAGGA;
hNotch3-R: AAAGTGGTCCAACAGCAGCTT;
hNotch4-F: GATAAAGATGCCCAGGACAACA;
hNotch4-R: GTCAGCAGATCCCAGTGGTTAC;
hNanog-F : GATCGGGCCCGCCACCATGAGTGTGGATCCAGCTTG;
hNanog-R : GATCGAGCTCCATCTTCACACGTCTTCAGGTTG;
hSox2-F : CCTCCGGGACATGATCAG;
hSox2-R : TTCTCCCCCCTCCAGTTC;
hJAGGED1-F: ATCCTCGAGAGCACCAGCGCGAACAGCAG;
hJAGGED1-R: ATCGAATTCCCCGCGGTCTGCTATACGAT;
hHEY1-F: ATCACCCACACATCGCACACCC
hHEY1-R: ACTAGGGGGCGCTCGCAAGG
mSox2-F:  TCAAGGCAGAGAAGAGAGTGTTTGC
mSox2-R: GAAGCGGAGCTCGAGACGGG
hGAPDH-F : ACCCAGAAGACTGTGGATGG;
hGAPDH-R:  TCTAGACGGCAGGTCAGGTC;
mHPRT-F: CACAGGACTAGAACACCTGC;
mHPRT-R: GCTGGTGAAAAGGACCTC.

**Microarray Data Acquisition and Analysis:**
RNA was isolated with miRNeasy kit (Qiagen) and quantified by Nanodrop 8000 Spectrophotometer (Thermo Scientific, Wilmington) and quality verified by RNA 6000 Nano kit (Agilent, Santa Clara, CA) on a Bioanalyzer 2100 and expressionanalysis used the Illumina platform (see Supplemental Methods). Biotinylated cRNA was prepared using Illumina TotalPrep RNA Amplification Kit (Ambion, Inc., Austin, TX) per manufacturer from 400ng total RNA. Samples were added to the Beadchip after randomization using a randomized block design to reduce batch effects. Hybridization to the Sentrix Human-HT12 Expression BeadChip (Illumina, Inc., San Diego, CA), washing and scanning were per Illumina BeadStation 500 manual (revision C). Microarray data analysis used Illumina GenomeStudio software.

**Microarray data processing and normalization:**
Microarray data processing and analysis performed used the R language and environment for statistical computing version 2.13 and Bioconductor version 2.8. Bead-summary expression data for the Illumina HumanHT-12 v4 BeadChip were normalized to correct for differences in expression within and between chips using the variance stabilization and normalization (vsn) method as implemented in the bead array R package version 2.2.0. Data for MDA-MB-231 and DT22 cell lines were separately normalized. The IlluminaHumanv4.db package version 1.10.0 was used to obtain probe mappings to official gene symbols. If multiple probes correspond to the same gene, the probe with the highest variance was used. In this way, the expression matrix was reduced so that each expression value corresponds to a single gene annotated by the official gene symbol.

**Defining a NOTCH target gene set:**
A NOTCH targets gene set was defined by GSI washout of a metastatic MDA-MB-231 variant treated with 10μM DAPT for 48 hrs, washed X 3, and cultured four more hrs in 10 mg/ml of cyclohexamide. Trizole extracted RNA was reverse transcribed and used with a custom TaqMan RT-PCR array cards for the candidate NOTCH target genes. Genes showing an upregulation of at least 1.5 fold in response to GSI washout were considered target genes.

**Gene Set Enrichment Analysis:**
MDA-MB-231 lines with discrete metastatic tissue tropisms have been used to define gene expression signatures for lung, bone, or brain metastasis (Bos et al, 2009; Kang et al, 2003; Minn et al, 2005). All three metastasis signatures were compared with the CD24 low and negative expression profiles. For the lung and brain metastasis gene signatures, the 18-gene and 17-gene versions used in clinical outcome analysis (Bos et al, 2009; Minn et al, 2005) were used, respectively. Probe identifiers were mapped to the official gene symbol. Only genes in the metastasis signatures that showed upregulation compared to parental MDA-MB-231 were included in the GSA of $CD44^+CD24^{low+}$and $CD44^+CD24^{neg}$ populations. For the embryonic stem cell signatures (Li YQ, 2010), the published gene lists were used and mapped to official gene symbols. We examined the signatures characteristic of human embryonic stem (hES) cells (Liu at al, 2007), of genes whose promoters are bound and activated by Nanog, Oct4 and Sox2 (NOS targets) in hES, and of the subset of NOS targets encoding transcriptional factors (NOS TFs) (Liu et al 2006; Li YQ, 2010). The gene signature from ES, NOS-targets, NOS-TFs were compared with the genes expressed in $CD44^+CD24^{low+}$ and $CD44^+CD24^{neg}$ populations. NOTCH targets gene set was identified by comparing genes expressed before and after GSI washout of a metastatic MDA-MB-231 variant. NOTCH target genes upregulated after GSI withdrawal were compared with those expressed in $CD44^+CD24^{low+}$and $CD44^+CD24^{neg}$ populations. Finally, genes differentially expressed in other stem cell enriched populations from cell lines and/or primary tumors (Dalerba et al, 2007; Frank et al, 2010; O'Brien et al, 2009) were separated into up- and down-regulated gene sets based on the published fold-change differences and also examined for enrichment in our $CD44^+CD24^{low+}$and $CD44^+CD24^{neg}$ populations.

These collections of gene signatures were used in Gene Set Analysis (GSA) as implemented in the GSA R package version 1.03. For GSA, a two-class paired comparison between and $CD44^+CD24^{low+}$ and $CD44^+CD24^{neg}$ cells using the maxmean method and re-standardization based on all genes in the microarray data set was used. Gene sets showing positive or negative enrichment were deemed significant if the false discovery rate and nominal p-value were less than 0.05 using 1000 permutations. GSA was performed separately for the MDA-MB-231 subpopulations and the DT-22 subpopulations.
